# Not Only Mutations Matter: Molecular Picture of Acute Myeloid Leukemia Emerging from Transcriptome Studies

**DOI:** 10.1155/2019/7239206

**Published:** 2019-07-30

**Authors:** Luiza Handschuh

**Affiliations:** Institute of Bioorganic Chemistry Polish Academy of Sciences, Poznan, Poland

## Abstract

The last two decades of genome-scale research revealed a complex molecular picture of acute myeloid leukemia (AML). On the one hand, a number of mutations were discovered and associated with AML diagnosis and prognosis; some of them were introduced into diagnostic tests. On the other hand, transcriptome studies, which preceded AML exome and genome sequencing, remained poorly translated into clinics. Nevertheless, gene expression studies significantly contributed to the elucidation of AML pathogenesis and indicated potential therapeutic directions. The power of transcriptomic approach lies in its comprehensiveness; we can observe how genome manifests its function in a particular type of cells and follow many genes in one test. Moreover, gene expression measurement can be combined with mutation detection, as high-impact mutations are often present in transcripts. This review sums up 20 years of transcriptome research devoted to AML. Gene expression profiling (GEP) revealed signatures distinctive for selected AML subtypes and uncovered the additional within-subtype heterogeneity. The results were particularly valuable in the case of AML with normal karyotype which concerns up to 50% of AML cases. With the use of GEP, new classes of the disease were identified and prognostic predictors were proposed. A plenty of genes were detected as overexpressed in AML when compared to healthy control, including* KIT*,* BAALC*,* ERG*,* MN1*,* CDX2*,* WT1*,* PRAME,* and* HOX* genes. High expression of these genes constitutes usually an unfavorable prognostic factor. Upregulation of* FLT3* and* NPM1* genes, independent on their mutation status, was also reported in AML and correlated with poor outcome. However, transcriptome is not limited to the protein-coding genes; other types of RNA molecules exist in a cell and regulate genome function. It was shown that microRNA (miRNA) profiles differentiated AML groups and predicted outcome not worse than protein-coding gene profiles. For example, upregulation of* miR-10a*,* miR-10b*, and* miR-196b* and downregulation of* miR-192* were found as typical of AML with* NPM1* mutation whereas overexpression of* miR-155* was associated with* FLT3*-internal tandem duplication (*FLT3*-ITD). Development of high-throughput technologies and microarray replacement by next generation sequencing (RNA-seq) enabled uncovering a real variety of leukemic cell transcriptomes, reflected by gene fusions, chimeric RNAs, alternatively spliced transcripts, miRNAs, piRNAs, long noncoding RNAs (lncRNAs), and their special type, circular RNAs. Many of them can be considered as AML biomarkers and potential therapeutic targets. The relations between particular RNA puzzles and other components of leukemic cells and their microenvironment, such as exosomes, are now under investigation. Hopefully, the results of this research will shed the light on these aspects of AML pathogenesis which are still not completely understood.

## 1. Introduction

Acute myeloid leukemia (AML), the most frequent leukemia in adults, is a severe myeloproliferative disorder with the high risk of relapse and high mortality rate [1, 2]. Random genetic alterations sequentially acquired by hematopoietic stem and progenitor cells disrupt hematopoiesis by differentiation blockades, uncontrolled growth and proliferation, and inhibition of apoptosis [3]. Immature, partially differentiated blast cells with self-renewal capacity first accumulate in bone marrow (BM) and then infiltrate peripheral blood (PB) and organs, impairing their functions [4]. Despite similar symptoms, blast morphology, and clinical implications, AML is a very heterogeneous disease presenting a wide spectrum of subtypes with different molecular features and outcomes [5, 6]. A number of chromosomal rearrangements and small mutations have been detected in AML and associated with the pathogenesis, diagnosis, and prognosis of the disease [5, 7, 8]. AML heterogeneity is also reflected in its classification, first established in 1976 by French-American-British (FAB) cooperative groups, based on morphological and cytochemical criteria [9] and revised nine years later [10]. In 2001, an alternative classification system, combining the leukemic cell lineage and maturation stage with genetic aberrations, was proposed by World Health Organization (WHO) and improved in 2008 and 2016 [11–13].

**Figure 1:**
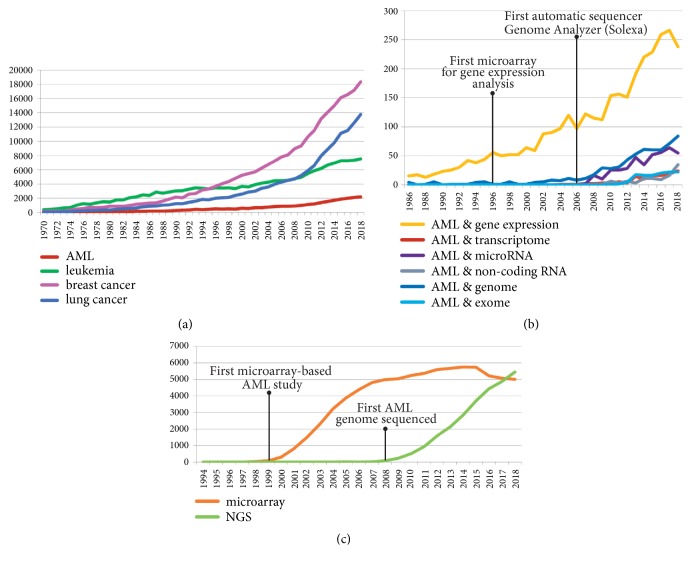
The number of publications found in PubMed, devoted to (a) AML, leukemia, and the two most common human cancers; (b) transcriptome and genome-based AML studies; (c) two the most common high-throughput technologies used in AML studies, microarrays, and next generation sequencing (NGS). The search terms and exact numbers of publications are noted in Supplementary Table 1.

**Figure 2:**
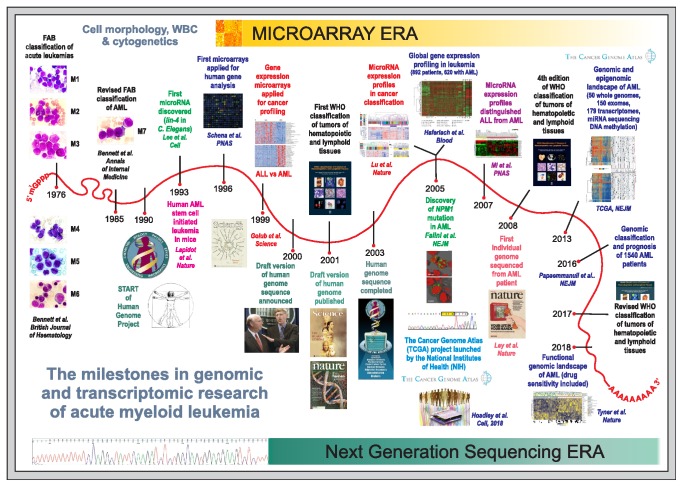
The milestones in genomic and transcriptomic research of acute myeloid leukemia. A symbolic mRNA molecule serves as a timeline on which the most important papers and events are marked, starting from the first FAB classification of AML in 1976 [9] and its revised version published in 1985 [10]. The microscopic images of M1-M7 FAB AML types come from the private collection of Prof. John M. Bennett and were used thanks to the courtesy of the Professor. The original pictures from the following publications were used with the permission of the authors and magazine publishers: Schena et al., PNAS 1996 [47] (Copyright 1996 National Academy of Sciences); Golub et al., Science 1999 [20] (reprinted with permission of AAAS); Lu et al., Nature 2005 [51] (reprinted by permission from Springer Nature, Nature, Copyright 2005); Falini et al., NEJM 2005 [52] (Copyright 2005 Massachusetts Medical Society, reprinted with permission from Massachusetts Medical Society); Haferlach et al., Blood 2005 [53] (reprinted by permission from American Society of Hematology, Copyright 2005); Mi et al. PNAS 2007 [54] (Copyright 2007 National Academy of Sciences); TCGA paper from NEJM 2013 [23] (Copyright 2013 Massachusetts Medical Society, reprinted with permission from Massachusetts Medical Society); Tyner et al. Nature 2018 [25] (reprinted by permission from Springer Nature, Nature, Copyright 2018); Hoadley et al., Cell 2018 [55] (reprinted by permission from Elsevier, Cell, Copyright 2018). Two Nature journal covers were reprinted by permission from Springer Nature, Nature, Copyright 2001 and 2008. Two Science journal covers, from 1999 and 2001, were reprinted with permission of AAAS. WHO publication cover images were reproduced with permission from Jaffe, E.S., Harris. N.L., Stein, H., Vardiman, J.W., Eds. WHO Classification of Tumours, Pathology and Genetics of Tumours of Haematopoietic and Lymphoid Tissues, IARC, Lyon, 2001 [11]; Swerdlow, SH, Campo, E, Harris, NL, Jaffe, ES, Pileri, SA, Stein, H, Thiele, J, Vardiman, JW. World Health Organization Classification of Tumours of Haematopoietic and Lymphoid Tissues. IARC, Lyon, 2008; Swerdlow, SH, Campo, E, Harris, NL, Jaffe, ES, Pileri, SA, Stein, H, Thiele, J, Arber DA, Hasserjian RP, Le Beau MM, Orazi A, Siebert R. World Health Organization Classification of Tumours of Haematopoietic and Lymphoid Tissues, revised 4th edition. IARC, Lyon, 2017. The photograph of Francis Collins and Craig Venter, made by Chuck Kennedy in 2000 (krtphotos001229) was used with the license of Newscom (https://www.newscom.com). Vitruvian man image was downloaded from Wikimedia Commons under a free license (https://commons.wikimedia.org/wiki/File:Vitruvian_man.jpg). Graphics representing the Human Genome Project (HGP) were used thanks to the courtesy of National Human Genome Research Institute ((NHGRI, https://www.genome.gov). The TCGA logo was used with the permission of National Cancer Institute (NCI, https://www.cancer.gov).

Although AML accounts for not more than 1% of all cancer diseases, it belongs to one of the most extensively studied human tumors, which has been confirmed by the ever-growing number of scientific reports (Figure 1(a)). In the Cancer Genome Atlas (TCGA), a landmark cancer genomics program (https://www.cancer.gov/tcga), AML is one of 33 cancer types collected hitherto, being represented by 200 cases. Availability of the tumor cells, which can be easily and in extensive amounts extracted from BM aspirates or even PB, makes AML a perfect model for cancer studies. In AML, the existence of a cancer stem cell was first demonstrated, proving the rightness of the tumor stem-cell concept [14–16]. Since then, our knowledge about cancer stem cells started to increase [6, 17]. Progress in the development of high-throughput methods such as microarrays and next generation sequencing (NGS) advanced our understanding of AML and other cancers [18, 19] (Figures 1(b) and 1(c)).  Figure 2 presents the timeline and milestones of AML research intertwined with the milestones of the Human Genome Project (HGP). The first application of global gene expression profiling (GEP) for cancer classification was demonstrated in 1999 on the example of two acute leukemias arisen from different lineages, myeloid (AML) and lymphoid (acute lymphoblastic leukemia, ALL) [20]. The first cancer genomes sequenced derived from AML patients [21, 22]. In 2013, TCGA Research Network published the sequences of 50 whole genomes and 150 exomes of AML patients [23]. Three years later, targeted resequencing of 111 genes in 1540 AML patients revealed more than 5 thousands of driver mutations [24]. In 2018, functional genomic landscape was drawn based on the exome sequencing, gene expression, and the analysis of* ex vivo* drug sensitivity in a cohort of over 500 AML patients [25]. Genome-wide studies revealed that the number of driver mutations in AML (on average, 13 somatic variants per patient) is lower than in solid tumors [23, 25]. New AML entities of diagnostic and prognostic significance have been identified and potential therapeutic targets have been indicated [26, 27]. Despite the tremendous effort put into research, AML (except for acute promyelocytic leukemia, APL) still lacks effective medical treatment [28, 29]. However, some promising therapeutic strategies are currently under investigation [30].

Despite the fact much attention has recently been devoted to genetic alterations occurring in AML, it should be remembered that the state of the cell is largely reflected by its transcriptome, which is the product of genomic activity. In fact, transcriptome-level analyses preceded whole genome sequencing and still serve as supplementary approaches in AML studies. In this review, I tried to show to what extent deeper insight into AML transcriptomes helped to unravel the mysteries of the disease.

## 2. The Early Beginnings: Studies of Single Protein-Coding Genes

At the turn of the 1980s and 1990s, more and more reports documenting gene expression in AML started to appear. Expression of several protooncogenes, encoding transcription factors (*MYC*,* MYB*, and* FOS*) and tyrosine kinases (*ABL1*,* FES*,* KIT*, and* PIM*) with essential roles in the regulation of hematopoiesis, cell proliferation, differentiation, cell cycle, and apoptosis was demonstrated in AML cells extracted from patients [31–34]. Erythroid progenitors and HEL erythroleukemia cells presented amplification of another transcription factor involved in cell proliferation,* E2F1* [35]. Overexpression of* RUNX1* gene, previously known as* AML1* or* CBF2A*, regulator of hematopoiesis, particularly of myeloid lineage, suppressed granulocytic differentiation and stimulated cell proliferation in murine cells [36]. Carow et al. [37] showed that expression of the hematopoietic growth factor receptor-encoding* FLT3* gene was not limited to normal stem/progenitor cells but was even elevated in leukemic blasts. In AML M2 with t(8;21), high percentage of CD34+ cells was correlated with high expression of* CD34* gene [38].* BMI1*, member of the polycomb complex, implicated in maintenance of normal and leukemic stem cells, was found to be expressed in AML M0 at a higher level than in other AML subtypes [39]. High expression of some genes was associated with adverse AML prognosis, e.g.,* WT1* [40],* MN1* [41],* BAALC* [42],* ERG* [43], and* EVI1* (ecotropic viral integration site 1, at present known as* MECOM*, from* MDS1* and* EVI1* complex locus) [44]. Genes which were renamed within the last years are listed in Table 1, together with the most commonly used abbreviations.

## 3. Microarray-Based Gene Expression Profiling: A Tool for Disease Classification

Technological progress enabled transition from single gene analysis to whole transcriptome scale at the end of the previous millennium [45]. The milestone was the invention of the microarray, chip-format tool which allowed for simultaneous analysis of thousands of genes in one test [46, 47]. Golub et al. [20] and Alizadeh et al. [48] were the first who showed that global gene expression profiling (GEP) could be a tool for cancer research and classification. Each group used a different type of microarrays: Golub et al. used commercially available high-density GeneChips made of short oligonucleotides synthesized in situ (Affymetrix) whereas Alizadeh et al. [48] constructed their own cDNA array, Lymphochip, dedicated to analysis of gene expression in normal and malignant lymphocytes. Golub et al. [20] proved that the distinction between two acute leukemias, AML, and ALL could be performed in a single test. Out of 6,817 human genes measured, expression of 50 genes was selected as the most closely correlated with AML-ALL class distinction. The 50-gene predictor was successfully validated in an independent collection, including samples from PB instead of BM, samples from childhood AML patients and samples collected by various laboratories. Among the most informative genes overexpressed in AML were known genes encoding cell surface proteins, e.g.,* CD33* and* CD11c* (currently* ITGAX*), and transcription factors, including* HOXA9* oncogene, whose high expression level was noted in AML patients with poor outcome. In fact,* HOXA9* seemed to be a single gene capable of predicting treatment failure in AML. The study revealed also novel AML markers, such as gene coding for leptin receptor with antiapoptotic activity, or zyxin gene encoding protein with cell adhesion function. The above results showed the power of GEP in disease classification and class discovery and encouraged other investigators to implement DNA microarray technology in their laboratories.

## 4. Protein-Coding Transcriptomes of Cytogenetically Defined AML Subtypes

Distinction between AML and other hematologic disorders seemed to be trivial. The question appeared whether gene expression profiles could successfully differentiate AML subgroups with cytogenetic and genetic abnormalities. Starting from AML with the most common chromosomal aberrations, different authors demonstrated that each of AML subgroups possessed its own gene expression signature and could be easily distinguished from one another. Below, the exemplary studies are described in more detail.

Virtaneva et al. [49] compared AML with isolated trisomy 8 (+8) to cytogenetically normal AML (CN-AML) and revealed fundamental biological differences between these two types of the disease. Common feature of both AML types was downregulation of genes encoding hematopoietic transcription factors (*STAT4*,* FUS*,* MCM3*, and* MCM5*) and myeloid markers (*ELANE* and* MPO*) in comparison to normal* CD34*+ bone marrow cells. Genes encoding complement factor D (*CFD*), proteins involved in cell growth and differentiation (*NDRG1* and* BTG1*), transcription factors* KLF6* and* ATF3*, and transcription coactivator* TAF10* were upregulated in both AMLs in relation to control* CD34*+ fraction. Two the most differentiating genes between AML +8 and CN-AML were* MLLT2* (present* AFF1* (*AF4/FMR2* Family Member 1), upregulated in CN-AML) encoding regulator of transcription and chromatin remodeling, and* FABP5* (upregulated in AML +8), encoding protein involved in fatty acid metabolism. Unsurprisingly, AML +8 blasts presented general overexpression of genes encoded on chromosome 8. The effect of genomic gains and losses on expression levels of genes located within the affected regions was further confirmed in a study of AML with trisomy 8, 11, 13, monosomy 7, and deletion 5q [50]. Surprisingly, in a study of Virtaneva et al. [49] protooncogene* MYC*, also encoded on chromosome 8, was downregulated in AML +8. On the one hand, in comparison to CN-AML, decreased expression level of proapoptotic genes (e.g.,* CRADD*,* BAD*) was noted for AML +8 whereas* TP53* gene, encoding tumor suppressor and also apoptosis inducer, was increased in AML +8. On the other hand, only CN-AML showed upregulation of antiapoptotic gene* DAD1*. Therefore, the authors suggested different mechanisms of cell death escape for the two studied leukemia types and associated it with AML +8 resistance to cytarabine-based chemotherapy, which should induce apoptosis [49].

AML subtypes with three reciprocal rearrangements, t(8;21)(q22;q22), inv(16)(p13q22), and (15;17)(q22;q12), corresponding to the morphological FAB subtypes M2, M4eo, and M3/M3v, respectively, were the subject of research of Schoch et al. [56]. Principal component analysis (PCA) of microarray data, performed with the use of 1000 most informative genes, clearly separated AML samples according to chromosomal aberration. The minimal set of 13 genes (*PRKAR1B, GNAI1, PRODH, CD52, KRT18, CLIP3, CLU, PTGDS, HOXB2, CLEC2B, CTSW, S100A9, and MYH11*) was sufficient to distinguish one AML subtype from another on the basis of gene expression solely. Expression levels of 36 genes enabled accurate classification of all three studied AML subtypes. Another set of 82 genes allowed for distinction of M3 and M3v, two phenotypically different AML types with t(15;17). In addition, the study showed that AMLs with alterations involved core binding factor (*CBF*) complex, t(8;21) and inv(16), were more related to each other than to AML with t(15;17). The authors explained the overexpression of* MYH11* in inv(16) and* RUNX1T1* (former* ETO*) in t(8;21) as a consequence of high expression of fusion transcripts affecting these genes. A new marker of t(8;21) was identified by Debernardi et al. [57], who found that a level of transcription factor-coding gene,* POU4F1* (former* BRN3A*), was 43-fold higher in t(8;21) AML than in other AML samples.

Verhaak et al. [58], by gene expression analysis in two independent cohorts of AML patients under 60, each exceeding 200 cases, perfectly distinguished three favorable cytogenetic AML subtypes, t(8;21), t(15;17) and inv(16). For AML with* NPM1* or* CEBPA* mutations, GEP-based classifiers were less accurate. The distinction of AML with other mutations (e.g.,* FLT3* and* RAS*) and aberrations (11q23, -5/5q-, -7/7q-, abn3q, and t(9;22)) was not possible with the use of GEP. Nevertheless, for abn3q, the most discriminative gene was* MECOM*, encoding an oncogenic transcription factor often involved in 3q26 abnormalities, and in 7(q) almost all differentially decreased genes were located on chromosome 7.

One of the most challenging tasks was to find unique features characterizing cytogenetically normal AML (CN-AML or NK-AML from normal karyotype AML) which accounts for 40-50% of all AML cases. Debernardi et al. [57] were the first who attempted to do that. Although the sample size was not large (28 adult AML samples, including 10 NK-AML) and NK-AML revealed higher variability than AML with translocations, the authors found NK-AML could be separated from AML samples with chromosomal rearrangements based on the expression levels of certain members of the class I homeobox A and B gene families, which were low or undetectable in AML with (t(8;21), t(15;17), and inv(16)). In NK-AML, expression level of 10 genes was extremely increased:* HOXA4, HOXA5, HOXA9, HOXB2, HOXB3, HOXB5, HOXB6, *and* HOXB7*, and two members of TALE family,* MEIS1* and* PBX3*. While overexpression of* HOXB* genes was unique for NK-AML, the upregulation of the remaining 5 genes was shared with 11q23 AML where* MLL* (mixed lineage leukemia) gene, now renamed to* KMT2A* (Lysine Methyltransferase 2A), was fused with different partners. High expression of some homeobox genes (e.g.,* HOXA3 *and* HOXB6*) was later found as typical of hematopoietic stem cells (HSCs) [59].

In 2004, two remarkable papers, identifying not only known but also new molecular AML subtypes through global GEP, were published in the same issue of the New England Journal of Medicine [60, 61]. Bullinger et al. (2004) [60] who analyzed 116 adult AML samples with the use of cDNA microarrays, found that hierarchical clustering with over 6 thousands of the most varied genes divided all AML samples into two main clusters. Out of the cytogenetic groups, only t(15;17) (APL) generated one condense subcluster. To enable biological insight into AML pathogenesis, group-specific gene expression signatures were established and functionally characterized. Signature specific for APL included genes related to hemostasis (*PLAU, SERPING1, ANXA8,* and* PLAUR*), resistance to apoptosis (*TNFRSF4, AVEN,* and* BIRC5*), impairment of retinoic acid-stimulated cell differentiation (*TBL1X, CALR,* and* RARRES3*) and resistance to chemotherapy (*CYP2E1, EPHX1,* and a group of metallothionein (*MT*) genes).* MLLT4* (present* AFDN*, Afadin), one of* KMT2A* fusion partners, was among the genes with unique expression profile in t(8;21), which suggested similar mechanism of pathogenesis with t(6;11). High expression of* NT5E*, observed in inv(16), was correlated with resistance to cytarabine. Interestingly, high expression of homeobox genes (*HOXA4, HOXA9, HOXA10, PBX3, *and* MEIS1*) was detected in AML specimens with not only normal but also complex karyotypes. Within NK-AML, Bullinger et al. [60] distinguished two distinct groups: one, where* FLT3* aberrations and FAB subtypes M1 and M2 prevailed, and the second one, where FAB M4 and M5 subtypes were more common. Of note, patients classified to those groups had different outcomes. For AML with complex karyotype, AML with* KMT2A* partial tandem duplications, and AML +8, it was impossible to find statistically significant unique gene expression signatures. Valk et al. [61] who analyzed 285 AML patients using Affymetrix GeneChips, identified 16 AML groups with distinct gene expression profiles. Some of them were composed of AML samples with known cytogenetic aberrations: t(18;21), t(15;17), and inv(16).* RUNX1T1* gene, which is a* RUNX1* fusion partner, was the most discriminative gene for AML with t(8;21). Overexpression of* MYH11* was the most discriminative feature of inv(16), which produces* CBFB-MYH11* fusion gene. Simultaneous downregulation of* CBFB* observed in this subtype could be explained by e.g., negative regulation of a wild-type* (wt) CBFB* allele by the fusion transcript. For APL, growth factor-coding genes were the most discriminative (hepatocyte growth factor (*HGF*), macrophage-stimulating 1 growth factor (*MST1*), and fibroblast growth factor 13 (*FGF13*)). However, AMLs with 11q23 were segregated into two separate clusters and partially scattered among all samples studied. Also NK-AML samples were divided into several clusters. The observed heterogeneity could be at least partially explained by the presence of particular mutations and different outcomes.

Further AML transcriptome studies, performed on independent patient cohorts, usually confirmed earlier research, reporting partially overlapping gene expression signatures. However, each study delivered a portion of new information, which deepened our knowledge about AML pathogenesis. Gutierrez et al. [62] performed hierarchical clustering of BM samples from 43 adult AML patients, based on the expression of over 5 thousand genes. Four distinct clusters they obtained corresponded to AML with inv(16), monocytic AML, APL and other AML samples which included NK-AML. The authors developed a minimal 21-gene predictor which classified each sample to appropriate group with 100% accuracy. Its efficiency was then confirmed with an independent AML sample set. APL samples, which formed the most condense group among all samples studied, revealed high expression of several growth factors and other signaling proteins, e.g.,* HGF, FGF13, MST1, VEGFA, IGFBP2,* and* FGFR1*. Contrary, overexpression of* HOX* family members (*A5, A6, A7, A9, A10, B2, B5, *and* B7*), including genes encoding TALE proteins (*MEIS1*;* PBX3*), and histone proteins was shared by all non-APL leukemias. Increased level of* MYH11* expression and downregulation of* CBFB* and* RUNX3* genes were noted specifically for AML with inv(16). In monocytic leukemia,* CSPG2*, other adhesion molecules such as the lectins* CLECSF6*,* CLECSF12, SIGLEC*7 and* FCN1* were upregulated compared to remaining AMLs. The remaining AML samples presented more heterogeneity, which was reflected by the existence of two subclusters, one with overexpression of genes encoding hematopoietic serine proteases, present in azurophil granules of neutrophilic polymorphonuclear leukocytes (*AZU1*, azurocidin 1,* ELANE* (previous* ELA2*), elastase,* PRTN3*, proteinase 3, and* CTSG*, cathepsin G), second with upregulation of* CD34* antigen, reflecting an early maturation arrest and lack of granulocytic differentiation.

AML M3 was extensively studied by Payton et al. [63] who compared the malignant promyelocytes from APL patients to leukemic cells collected from other AML subtypes and to promyelocytes, neutrophils and* CD34*+ cells extracted from healthy bone marrow donors. The identified “M3-specific dysregulome” was composed of 510 genes and many of them exhibited dramatic differences in expression level comparing to other AML subtypes or normal promyelocytes. For example,* GABRE, FGF13, HGF, ANXA8*, and* PGBD5* were the most overexpressed genes whereas* VNN1, MS4A6A, P2RY13, HK3*, and* S100A9* the most underexpressed genes in M3 vs. other AMLs. 33 genes selected from the identified signature were validated by another high-throughput digital technology (nCounter; NanoString), capable of detecting as little as 0.5 fM of a specific mRNA and measuring up to 500 genes in a multiplex reaction. The authors demonstrated nCounter reproducibility and applicability as a tool for biomarker analysis when limited amounts of clinical material are available. 33 genes validated by NanoString assay were also enriched in an independent AML dataset of 325 samples, and APL mouse model, but, notably, not in a cell line expressing* PML-RARA* fusion gene.

One of the most impressive microarray-based studies was that of Haferlach et al. [53] who analyzed almost 900 patients with leukemia, including 620 with AML, with the use of Affymetrix GeneChips and support vector machine (SVM) model. The authors identified 13 separate leukemia types, including 6 within AML. Some of them, e.g., AML with t(8;21) and with t(15:17), could be classified with 100% specificity and 100% sensitivity based on the expression profile of 100 genes per group. The overall prediction accuracy of 95.1% was achieved. The misclassification occurred mainly in subgroups with a low sample number or high intragroup heterogeneity. The largest and the most heterogeneous subgroup was AML with normal karyotype, which cosegregated with AML with less common cytogenetic aberrations classified as “other”. Here, AML samples with different fusion partners of* KMT2A* gene were included. Haferlach et al. [64] showed that APL was not only distinct from other AML subtypes in the matter of gene expression, but two M3 phenotypes, one with heavy granulation and bundles of Auer rods and AML M3 variant (M3v) with non- or hypogranular cytoplasm and a bilobed nucleus, could be discriminated based on gene expression signatures.

The largest GEP study in hematology and oncology was conducted thanks to international collaboration within the European Leukemia Net. In 2010, Gene Expression Profiling Working Group directed by Torsten Haferlach published the results of analysis of 3,334 samples collected from leukemia (including 542 AMLs) and MDS patients by 11 laboratories across three continents [65]. Apart from European ones, laboratories from the United States, and one from Singapore joined the program. The main conclusion was GEP was a robust technology for the diagnosis of hematologic malignancies with high accuracy. According to the authors, GEP had invaluable application potential and was vulnerable to standardization, outperforming more subjective methods such as cytomorphology and metaphase cytogenetics. To enable better molecular understanding of leukemias, the authors deposited the collected data into a publicly available domain.

In 2012, de la Blétière et al. [66] proved that AML cytogenetic subtypes could be successfully determined with the use of GEP, even in samples with low leukemic blast content or poor quality. With the use of Illumina Expression Bead-Chips, the authors first classified 71 good quality samples from a training set, representing APL, t(8;21)-AML, inv(16)-AML, or NK-AML with at least 60 percent of leukemic blasts. The optimal 40-marker gene classifier (10 markers per class, including previously described as well as newly discovered genes) was applied to 111 suboptimal AML samples with low leukemic blast load (from 2 to 59%) and/or poor quality control criteria. The overall error rate was 3.6%. All APL and t(8;21) samples were correctly classified, even those containing as low as 2 percent blasts. The worst result was achieved for inv(16). Surprisingly, poor sample quality did not affect classification. By the way, de la Blétière et al. [66] demonstrated reliability, robustness, and sensitivity of Illumina bead-based technology which seemed to be not worse than other, commercially or academically developed, microarray platforms used before.

## 5. Between AML and ALL: Acute Leukemia with* KMT2A* Rearrangements

In 2002, Armstrong et al. [67] showed that ALL with translocations involving the* KMT2A* gene (previously known as* MLL*) presented a unique gene expression profile, different from ALL and AML without* KMT2A* abnormalities. The core of this unique gene expression signature consisted of multilineage markers of early hematopoietic progenitors and* HOX* genes, which corresponds with the fact that* KMT2A* gene encodes histone lysine methyltransferase, a transcriptional coactivator regulating expression of genes (including* HOX*) during early development and hematopoiesis. Therefore, the authors proposed to distinguish a distinct leukemia entity termed “MLL”. Then, a common gene expression signature, enriched in homeobox genes (*MEIS1, HOXA4, HOXA5, HOXA7, HOXA9,* and* HIOXA10*), was determined for all acute leukemias with* KMT2A* fusion, irrespectively of their lineage (myeloid or lymphoid), by Ross et al. [68]. Similarly, Andersson et al. [69] associated childhood acute leukemias with* KMT2A* rearrangements with upregulation of homeobox genes (*HOXA10, HOXA4, MEIS1* and* PBX3*). In their study,* KMT2A*-positive AMLs were also enriched in genes involved in cell communication and adhesion, whereas some antiapoptotic genes (e.g., a tumor necrosis factor receptor,* TNFRSF21*) and tumor suppressor genes (*BRCA1; DLC1*) were downregulated in this AML subtype. Hierarchical clustering with a subset of genes encoding transcription factors showed that leukemic samples with* KMT2A* rearrangements grouped together, independently on lineage. Although* KMT2A* translocations are prevalent in infant and treatment-related leukemias, they also occur in adult leukemias that were studied by Kohlmann et al. (2005) [70] who wondered how the differing* KMT2A* partner genes influenced the global gene expression signature and whether pathways could be identified to explain the molecular determination of* KMT2A* leukemias of both lineages. The data analysis in both types of acute leukemias revealed t(11q23)/*KMT2A*-positive samples that were evidently distinct from other subtypes of the same lineage. As in the case of childhood leukemia, adult* KMT2A*-AML and* KMT2A*-ALL, despite a shared common gene profile, revealed also lineage-specific expression markers sufficient to segregate them according to their lineages, with no respect to the* KMT2A* fusion partner. The commonly overexpressed genes were obviously the homeobox genes and their regulators (*HOXA9, MEIS1, HOXA10, PBX3, HOXA3, HOXA4, HOXA5, HOXA7*),* NICAL* gene (present* MICAL*, encoding Microtubule Associated Monooxygenase),* RUNX2* transcription factor and* FLT3* gene. The common downregulated genes included TNF-receptor superfamily members (*TNFRSF10A* and* TNFRSF10D*), transcription factor* POU4F1*, tumor suppressor* ST18* or* MADH1* (present* SMAD1*), encoding a signal transducer and transcriptional modulator. Comparing to* KMT2A*-ALL, overexpression of* CEBPB*,* CEBPA*,* KIT*,* MADH2*,* MITF*,* FES* and* SPI1* (former* PU.1*) oncogenes, and* MNDA*, encoding the myeloid cell nuclear differentiation, was noted in* KMT2A*-AML. Summarizing their results, the authors concluded AML with t(11q23)/*KMT2A* and ALL with t(11q23)/*KMT2A* are rather distinct entities.

## 6. AML Risk Classification and Outcome Prediction

AML chemotherapy does not always lead to complete remission (CR). 20-50% AML patients are primarily resistant to induction therapy. Having this information at the time of diagnosis would facilitate treatment decision making. Taking into account the success of GEP in AML diagnosis and classification to particular disease subtypes, its application in prognosis prediction was only a matter of time. Correlation of* HOXA9* upregulation with poor AML outcome was reported by Golub et al. in their first microarray paper, devoted to ALL and AML classification [20]. Later, Andreeff et al. [71] demonstrated that many AML cases with intermediate and adverse prognosis, presented* HOX* expression levels similar to the levels observed in normal* CD34*+. Interestingly,* HOXA* genes could distinguish favorable vs. unfavorable cases, but only* HOXB* genes effectively distinguished intermediate from unfavorable AMLs. Despite the high coordination in* HOX* gene family expression,* HOXA9* seemed to be the best single predictor of overall survival (OS), disease-free survival (DFS) and response to therapy, confirming earlier results of Golub et al. [20].

AML outcome prediction was often matched with AML classification. Analysis of AML GEP-based clusters defined by Valk et al. [61] in the context of prognosis showed that three clusters, overlapping with inv(16), t(15;17), and t(18;21), were associated with good outcome. Patients classified to the cluster that common feature was* MECOM* overexpression had clearly worse outcome.

Two prognostically different NK-AML subgroups were also identified by Bullinger et al. [60]. A group with predominance of* FLT3* aberrations and FAB subtypes M1 and M2 presented shorter OS. High expression of* GATA2, NOTCH1, DNMT3A* and* DNMT3B* in this group suggested pathological impact of aberrant methylation. Genes deregulated in the second group, where FAB M4 and M5 subtypes were more common, were associated with granulocytic and monocytic differentiation, immune response and hematopoietic stem-cell survival (*VEGF*). Analysis of prognostically relevant genes led to the identification of 133-gene based prognostic signature.* FOXO1A*, encoding transcription factor involved in cell cycle arrest and apoptosis regulation, was one of the genes correlated with favorable outcome. Poor outcome was determined by overexpression of* HOX* genes and* FLT3* gene. Prognostic gene expression signature proposed by Bullinger et al. [60] was 2 years later applied to an independent cohort of 64 NK-AML patients below the age of 60 by Radmacher et al. (2006) [72]. GEP of the new sample set, performed with Affymertix GeneChips, different array technology than one that was used to establish the prognostic signature, allowed segregation of patients into 2 clusters with significantly different OS and DFS. Strong association between the outcome classification and* FLT3*-ITD status was observed: 67% patients with poor outcome were* FLT3*-ITD-positive. However,* FLT3*-ITD was present in almost 20% of patients from the good-outcome class, which indicated contribution of other prognostic determinants. Nevertheless, Radmacher et al. [72] not only validated the previous prognostic signature, but also developed a well-defined classifier, which might be applied to individual patients, with best accuracy to patients with normal cytogenetics and wt* FLT3*.

Application of gene expression microarrays for prediction of patient sensitivity to therapy was also demonstrated by Heuser et al. [73] and Tagliafico et al. [74]. Heuser et al. [73] identified gene expression profile that distinguished AML M0-M5 (excluding M3) patients with good or poor responses. Hierarchical clustering performed on a training set of 33 AML samples divided good responders into two clusters, which suggested the effect of determinants other than treatment. Interestingly, samples with the lowest level of myeloid cell maturation, corresponding to FAB subtypes M0 and M1, were equally distributed between clusters representing good and poor response. Over 30% of poor-response-associated genes, e.g.,* MN1, FHL1, CD34, RBPMS, LPAR6*, and* FLJ14054* gene (currently known as* NPR3*), were earlier described as overexpressed in hematopoietic stem or progenitor cells, particularly in the populations with the highest self-renewing capacity. Application of the identified gene expression signature to the test set of independent 104 AML samples enabled dividing them into two prognostic subgroups which correlated with the different response to induction chemotherapy. The accuracy of prediction was 80%. Tagliafico et al. [74] conducted a similar analysis, but their training set included 10 blast cell populations collected form AML patients and 6 AML cell lines with determined sensitivity to differentiation therapy. The identified prediction set, containing such genes as* MEIS1* and* MS4A3*, was then tested on the GEP datasets published by Valk et al. [61] and Bullinger et al. [60]. Despite a significant overlap in prognosis prediction, Tagliafico et al. [23] distinguished within the poor outcome groups described in original papers, a subgroup of patients (20-40%) which revealed sensitivity to maturation induction. From the practical point of view, it suggested that these patients could benefit from a differentiation therapy even though the initial prognosis was unfavorable.

Gene expression analysis of samples from 170 older AML patients (median age 65 years, all FAB subtypes except for M3), presented by Wilson et al. [75], showed the problem of response to therapy as even more complex. Hierarchical clustering divided patients into 6 groups with different rates of resistant disease, complete response, and DFS. Distribution of FAB subtypes and* NPM1* (but not* FLT3*-ITD) mutation differed significantly between clusters, but in only two clusters particular subtypes prevailed, e.g., one cluster almost exclusively consisted of monocytic leukemias (M4 and M5). Poor-risk clusters had lower WBC and blast counts whereas cluster with the best DFS and OS contained 75% of NK-AML and 78% samples with* NPM1* mutations. Each cluster was defined by a specific expression profile of the 50 most discriminating genes. For example, in a cluster with the poorest outcome, the authors observed upregulation of multidrug resistance genes (*ABCG2* and* ABCB1*, former* MDR1*), homeobox gene* PBX1*, which prevents myeloid differentiation, and* STK17* gene, encoding apoptosis regulator. Another poor outcome cluster revealed overexpression of genes connected with immunity (*IRF4, IL10RA, *and* MALT1*). The most favorable outcome cluster was characterized by overexpression of genes encoding proteins implicated in cell signaling (*IL12A*), promoting apoptosis (*CASP3 *and* LTBP1*), and leukemic transformation (*MEIS1, WT1,* and* FOXC1*), and downregulation of genes encoding major histocompatibility complex (MHC) proteins of class II.

Based on gene expression data from a training cohort of 163 AML patients collected by the German AML Coopertive Group, Metzeler et al. [76] elaborated 66 gene expression signatures to predict OS in CN-AML. Then, the signature was validated in two independent cohorts of 79 and 64 CN-AML patients from Europe and the United States, respectively. In all three cohorts, patients with low gene expression risk score had better outcome. Moreover, in multivariate analyses of validation cohort, the gene expression score proved to be a stronger prognostic factor than age, presence of* FLT3*-ITD, and* NPM1* mutation. The genes from the identified signature partially overlapped with the results of previous studies, e.g.,* TCF4, FHL1, CD109*, and* SPARC* genes, had been earlier associated with poor response to chemotherapy [73].

## 7. Looking for New Therapeutics

Transcriptome, as well as proteome, reflects the current cell status that dynamically evolves under the influence of various stimuli, e.g., therapeutic agents. GEP is a sensitive tool to detect changes in genome activity; therefore it can be applied to monitor minimal residual disease (MRD) and cancer cell reaction to novel compounds. AML treatment is challenging because resistance to therapy is quite common and even those patients who achieve CR are prone to relapse. GEP was widely applied for analysis of resistance mechanisms and efficiency of potential drugs. Kinase inhibitors in the treatment of AML with* FLT3*-ITD, correlated with negative prognosis, have been studied for a long time. In April 2017, staurosporine derivative PKC412 (midostaurin), a multikinase inhibitor, was approved by the US FDA for the treatment of newly diagnosed* FLT3*-mutant AML in combination with chemotherapy [77]. Activity of this compound was analyzed, inter alia, in human myelomonoblastic cell line MV4-11 carrying* FLT3*-ITD by Stölzel et al. [78] with the use of gene expression microarrays. Two versions of MV4-11 cells, sensitive and resistant, were compared prior to and after treatment. Significant downregulation of* TP53* and upregulation of* JAG1* was observed in resistant cells before and after treatment.* MCL1* and* KIT* genes were upregulated in resistant MV4-11 cells after incubation with PKC412. The authors concluded that resistance against PKC412 was mediated by antiapoptotic gene activation and proapoptotic signal decrease, with contribution of deregulation of genes involved in normal and malignant hematopoiesis.

Tavor et al. [79] studied gene expression response of the AML cell line U937 under treatment with the CXCR4-antagonist, AMD3100. CXCR4, a receptor for SDF-1 chemokine secreted by stromal cells, participates in the interactions of leukemic stem cells with the BM microenvironment, necessary for cell migration and disease progression. In addition the role of elastase, neutrophil serine protease synthesized during the transition of myeloblast to promyelocyte, was investigated. The authors did not observe changes in gene expression after treatment with anti-CXCR4 antibody or elastase inhibitor, but found AMD3100-induced suppression of the SDF-1/CXCR4 axis or elastase inhibited leukemic cell proliferation as well as activated genes involved in myeloid differentiation.

Other candidates for target therapeutics in AML treatment are in clinical trials. One example is pinometostat (EPZ-5676), a small-molecule inhibitor of DOT1L (histone methyltransferase disrupter of telomeric silencing 1-like). Pinometostat, considered for combination therapies of acute leukemias with* KMT2A* gene rearrangements, was proved to target DOT1L and reduce H3K79 methylation in adult AML patients with 11q23 translocations [80]. Another promising therapeutic strategy is directed against members of Hedgehog (HH) signaling pathway, which plays a role in embryonic cell development as well as in proliferation and maintenance of adult stem cells, including cancer stem cells [81, 82]. Comparing chemotherapy-sensitive and resistant cell lines, Queiroz et al. [83] indicated HH pathway as an essential component of myeloid leukemia MRD. Overexpression of HH pathway effectors,* GLI1* and* PTCH1,* followed by constitutive activation of HH signaling, was correlated with chemoresistant phenotype. The efficacy of a HH pathway inhibitor, glasdegib, which targets a smoothened protein (SMO), a G protein-coupled receptor interacting with PTCH1, was evaluated by Cortes et al. in AML and high-risk MDS patients who were not eligible for intensive chemotherapy [81]. At the end of 2018 glasdegib has been approved in the USA, under the name DAURISMO™, for use in combination with low-dose cytarabine for the treatment of newly diagnosed AML patients excluded from intensive induction chemotherapy due to age or comorbidities [84]. Another compound, GANT61, the inhibitor of GLI family proteins, was shown to specifically target the* CBFA2T3-GLIS2* fusion gene in pediatric AML [82]. The authors demonstrated that GANT61 treatment significantly reduced the expression level of* GLIS2* and a gene encoding bone morphogenic protein (*BMP2*). Posttreatment microarray-based gene expression analysis revealed downregulation of* CBFA2T3-GLIS2* target genes as well as genes required for cell cycle progression, cell proliferation, and epigenetic regulation. New AML therapies are still being elaborated. Currently, the US National Cancer Institute (NCI) supports 75 clinical trials for adult AML treatment (https://www.cancer.gov/about-cancer/treatment/clinical-trials/disease/adult-aml/treatment). It is impossible to provide even a brief summary of all of them in this work.

## 8. Pediatric AML: Distinct but Similar

AML in children is less frequent than in adults but reveals similar level of heterogeneity. In both age groups, similar chromosomal aberrations and mutations occur, though with different proportions. In children, CN-AML concerns only about 20% of all AMLs and the frequency of mutations is generally lower [85]. The power of GEP demonstrated on adult AML samples triggered the research of childhood AML. First, the 35-gene expression signature was shown to predict prognosis in pediatric AML [86]. Genes encoding cyclins and cyclin-dependent kinases required for cell cycle progression (*CDK6, CCND1, * and* CDC25A*), and* TRAF2* gene encoding a signal transducer activating* NFKB1*, showed higher expression in patients with poor outcome. The levels of* NFKBIA*, encoding* NFKB1* inhibitor, and* STK17B*, encoding serine/threonine protein kinase inducing apoptosis, were lower in patients with poor outcome.* STK17B* downregulation and* NFKB1* enhancement might explain why patients with adverse prognosis escaped from chemotherapy-induced apoptosis. An additional reason for the poor outcome could be increased cell cycle progression. Comparing pediatric AML patients with different FAB subtypes, the authors selected 213 probe set representing genes, whose expression correlated with FAB subtype. Both signatures, prognostic and diagnostic, shared only three genes (*TYMP, STK17B,* and* ATP6V0B*).

Ross et al. [68] compared gene expression in 130 pediatric and 20 adult AML samples with Affymetrix GeneChips. Some AML groups, namely t(15;17), t(8;21) and FAB M7, more frequent in children than adults, were clearly distinguished whereas AML with* CBFB/MYH11* fusion gene (inv(16)) and* KMT2A* chimeric fusion genes revealed more heterogeneity, indicating the existence of additional subgroups. Biology of the disease seemed to be similar, independently on age, as only minimal differences were observed in gene expression profiles between pediatric and adult AML samples containing the same lesions. The authors identified a set of class discriminating genes, which included genes specifically overexpressed in particular AML FAB types, e.g., AML M2, was characterized by increased expression of genes coding for cell surface antigens (*CD34*;* CD19*), proteins regulating developmental processes (*ROBO1, TWSG1, *and* PELI2*) and transcription factor* POU4F1*. Genes upregulated in AML M3, M4Eo and M7 encoded proteins reflecting particular stages of myeloid differentiation or lineage, for example,* HGF*,* MPO* and* CPA3* in M3,* CD52* and* CHI3L1* in M4Eo,* GP1BB* and* ITGA2B* in M7. The results concerning AML with* KMT2A* rearrangements were described above. What is interesting, Ross et al. [68] tested on their dataset the 35-gene prognostic signature described by Yagi et al. [86] and did not confirm its correlation with patient outcome. Instead, Ross et al. [68] selected another, small set of genes whose high expression correlated with poor outcome. This shows gene expression profile dependence on sample set, sample size, protocols and laboratory. However, another study of childhood leukemia, published by Andersson et al. [69], confirmed the results obtained by Ross et al. [68], presenting 77–86% overlap between the differentially expressed genes (DEGs).

Analysis of 237 pediatric AML cases with gene expression microarrays and double loop cross-validation method allowed for the selection of 75 probe sets, representing 59 unique genes, able to classify AML with the five most prevalent cytogenetic subtypes, constituting about 40% of pediatric leukemia [85]. Among the most discriminative genes were* WHAMMP3* and* ITM2A* (encoding membrane associated proteins) for* KMT2A*-rearranged;* RUNX1T1*,* IL5RA* and* POU4F1* for t(8;21);* MYH11*,* LPAR1* and* NT5E* for inv(16);* HGF*,* STAB1* and* FAM19A5* for t(15;17);* TP53BP2* (coding for p53-binding protein), and* DNAAF4* (encoding protein interacting with the estrogen receptors and the heat shock proteins) for t(7;12). The accuracy of the classifier, validated on two independent cohorts of patients, was equal to 92% and 99%. However, GEP had limited predictive value for AML cases with* NPM1*,* CEBPA*,* KMT2A*(-PTD),* FLT3*(-ITD),* KIT*,* PTPN11*, and* N/K-RAS* mutations, perhaps because of generally lower frequency of mutations in children than in adults.

## 9. AML in the Elderly

AML is a disease of older adults. Within age, not only the incidence of illness increases; elderly AML patients usually present worse outcome and weaker response to therapy. Rao et al. [87] reanalyzed clinically annotated GEP data from 425 de novo AML patients in the context of age. From this dataset, two age-related cohorts were selected: 175 young (<or= 45 years) patients and 144 elderly (>or= 55 years) patients. Indeed, both cohorts significantly differed in OS and DFS. This difference could be explained by unique pattern of deregulated signaling pathway found for older AML patients, who had a lower probability of E2F and PI3-kinase pathway activation but a higher probability of RAS, TNF, SRC, and EPI pathway activation. Thus, the authors concluded AML in the elderly represents a distinct biologic entity. The same conclusion was made by de Jonge et al. [88] who discovered the downregulation of the tumor suppressor gene* CDKN2A* in older AML patients with intermediate- and unfavorable prognosis.* CDKN2A* gene encodes a cyclin-dependent kinase inhibitor known as p16(INK4A) protein whose amount increases with physiologic ageing. The authors showed that p16-INK4A besides cytogenetic risk groups, was an independent OS prognostic parameter in older patients. The conclusion was that in the elderly, oncogenesis might be facilitated by a suppression of defense mechanisms, which usually protect older cells against cell and DNA damage [89].

## 10. Between MDS and AML

Myelodysplastic syndromes (MDS) are a group of clonal heterogenous hematologic malignancies frequent in the elderly, characterized by progenitor cell dysplasia, ineffective hematopoiesis and a high rate of transformation to AML [90]. Due to the not clearly defined boundaries between MDS and other myeloid disorders, establishing MDS diagnosis with conventional method is often problematic. Looking for a novel diagnostic strategy, Miyazato et al. [91] compared the transcriptomes of MDS with de novo AML and other bone marrow diseases, with the use of custom-made oligonucleotide microarrays. The hematopoietic stem-cell fractions were purified based on the expression of the surface marker* PROM1*, previously named* AC133* or* CD133*. The authors identified a small set of genes preferentially expressed in MDS (e.g.,* DLK1, TEC, *and* ITPR1*) or AML (e.g., genes encoding solute carrier (*SLC*) family members, opioid receptor delta 1 (*OPRD1*) and leptin receptor (*LEPR*)).

AML with dysplasia, which has a poor outcome with conventional chemotherapy, was studied by Tsustumi et al. [92]. The authors analyzed three AML subcategories with dysplastic morphology, AML with multilineage dysplasia (AML-MLD), MDS-related AML (MDS-AML), and therapy-related leukemia (TRL), and compared them with de novo AML without dysplasia. As in the study of Miazato et al., fractions of BM hematopoietic stem cells presenting CD133 antigen were selected for microarray-based transcriptome analysis. 56 genes displayed different expression levels between AML-MLD and MDS-AML. The genes preferentially expressed in AML-MLD comprise many genes encoding nuclear proteins, ubiquitination-related proteins, and* PF4* gene encoding platelet factor 4, a chemokine secreted by platelets and influencing BM environment. The same gene was overexpressed in AML-MLD also when compared to AML without dysplasia, suggesting the correlation of PF4 expression with AML-MLD. Distinction between MDS-AML and AML without dysplasia was possible with the use of 28 genes, including 9 shared within the 56-gene signature differentiating AML-MLD and MDS-AML. One of them,* LAPTM5* gene, encoding lysosomal transmembrane protein, was clearly upregulated in MDS-AML, being a candidate for novel marker for MDS-related leukemia. However, the gene signatures determined by Tsustumi et al. [92] were not perfect, which showed global gene expression analysis may be not adequate for AML subgroups with high intragroup heterogeneity and subtle intergroup differences.

## 11. Bone Marrow Microenvironment

The main attention of AML investigators was focused on leukemic blasts. However, it is well known that other factors, such as tumor microenvironment, contribute to disease progression. In hematological malignancies, the interplay of cancer cells and surrounding stroma is particularly important. BM microenvironment consists of a heterogeneous population of cells directly involved in hematopoiesis or supporting hematopoietic cell function, migration, adhesion, metabolism, and differentiation, e.g., by production of ligands and cell adhesion molecules [93]. The role of BM niche in AML has not been fully elucidated and is currently intensely studied [4, 93]. Experiments with the mouse models indicated that the BM microenvironment not only facilitates the leukemic cell growth but can even initiate leukemogenesis in healthy cells [94]. The expansion of a single dominant hematopoietic progenitor clone is favored in the aged BM microenvironment, which causes monoclonality and may contribute to higher rates of leukemia incidence with age [95]. Moreover, BM niche protects quiescent LSCs, being responsible for MRD and relapse. On the other hand, BM stromal cells reveal high level of plasticity and can also be affected by malignant cells [93, 96]. Therefore, the disease progression depends on the leukemia-microenvironment crosstalk. One of the best recognized interactions between leukemic blasts and stroma is directed by a transmembrane chemokine receptor CXCR4, highly expressed by leukemic cells, and CXCL12 protein secreted by BM stromal cells. CXCR4-CXCL12 binding promotes the homing, residence, and survival of leukemic cells in the BM [4]. Another interaction, between the integrin VLA-4, expressed by leukemic cells, fibronectin present in the extracellular matrix, and VCAM-1 on BM stroma, contributes to chemoresistance [4].

In 2018, Kumar et al. described how AML blasts transform the BM niche into a leukemia-promoting and normal hematopoiesis-suppressive microenvironment through a secretion of exosomes, small vesicles mediating cell-to-cell communication [96]. The authors demonstrated that AML-derived exosomes target stromal and endothelial cells in the BM niche. Using human-to-mouse AML graft models, they proved AML-derived exosomes caused changes in mice, similar to those induced by AML cells, i.e., reshaped the BM niche cell composition and modulated gene expression in stromal cells. Genes required for normal hematopoiesis and bone development, e.g.,* CXCL12*,* KITL* and* IGF1*, were downregulated whereas a hematopoiesis and osteogenesis suppressor,* DKK1*, was upregulated. Reduction of exosome secretion in AML cells delayed the disease progression.

One of the recent studies used a unique* ex vivo* model of growing leukemic cells on patients' own stroma (POS) derived in diagnosis (Dx), remission (Rm) and relapse (Rx) [97]. Compared to healthy mesenchymal stromal cells (MSCs), POS presented different morphology, larger cell size, reduced proliferation rate, slower expansion, and poor cell-cell contact. Coculture cross experiments revealed that POS preferentially supported proliferation of the same patient's AML cells, irrespective of the disease state POS was obtained in. The unique crosstalk between POS and AML cells was mediated by cytokines and chemokines, angiopoietin 1, secreted phosphoprotein 1, and SDF-1, encoded by* ANGPT1* (former Ang-1),* SPP1* (former* OPN*), and* CXCL12* genes, respectively. Compared to healthy MSCs,* SPP1* expression was higher in Dx/Rx and Rm POS whereas* ANGPT1* expression was upregulated in Dx/Rx POS and increased in the presence of AML cell. In contrast,* CXCL12* was decreased in Dx/Rx and Rm POS, which was associated by the authors with interruption in the CXCL12-CXCR4 signaling, and a consequent loss of hematopoietic progenitor quiescence and induced proliferation. Interestingly, POS demonstrated similar features in remission as in the active disease, which indicates the critical role of BM niche in relapse and treatment failure.

BM microenvironment-mediated protection of* FLT3*-ITD AML from tyrosine kinase inhibitors (TKIs) was recently reported by Chang et al. [98]. Drug resistance was a result of elevated expression of genes encoding cytochrome P450 enzymes, in particular* CYP3A4*, by BM stromal cells. Because CYP3A4 inhibitor reversed the protective effects of BM niche, the authors proposed a combination of* FLT3* TKIs with CYP3A4 inhibitors as a novel strategy to treat* FLT3*-ITD AML.

Passaro et al. [99], who studied the BM vasculature in AML using intravital two-photon microscopy, associated increased vascular permeability in the BM microenvironment with disease progression and poor treatment response. Transcriptome analysis of BM-derived endothelial cells via RNA-seq identified deregulation of genes involved in vasculature development, angiogenesis, and response to hypoxia.* Nox4* gene, encoding NAPDH oxidase, responsible for production of reactive oxygen species (ROS), activation of nitric oxide synthase 3 (NOS3), and release of nitric oxide (NO), was particularly upregulated. Increased NO level contributed to the vascular leakiness in AML-engrafted mice and was associated with poor prognosis in AML patients. Application of NO synthase inhibitors combined with standard chemotherapy restored normal vasculature and improved the treatment response, demonstrating the efficacy of combined leukemia-niche therapies.

The role of lymphocytes and other blood cells has long been neglected in the studies of AML. However, Le Dieu et al. found that the absolute number of T-cells circulating in PB of de novo AML patients, not belonging to malignant clones, was increased compared to healthy controls [100]. Activation of T-cells might reflect a response to growth signals present in a local microenvironment. GEP of CD4+ and CD8+ T-cells from AML patients and healthy volunteers revealed global differences in transcription pattern, with little similarities to T-cells of CLL patients. Particularly, genes associated with the actin cytoskeleton and cellular polarization were deregulated in AML T-cells. According to the authors, T-cell aberrant activation leads to their dysfunction and impaired immune response, which is not a sufficient weapon against the leukemic blasts. Here, a rationale to apply immunomodulatory drugs appears.

## 12. Discovering the Power of Small Molecules: miRNA Expression Profiling

The discovery of regulatory role of RNA in cell and organism development completely changed our understanding of biology and at least partially explained the paradox that is the large size of mammalian genomes of which only a small percentage are the protein-coding genes [101–103]. Among small regulatory RNAs, microRNAs (miRNAs), termed due to their small size (18-23 nt), are best recognized [104, 105]. The function of miRNAs in gene expression regulation (usually repression), controlling cell fate and normal developmental processes as well as oncogenesis, is well-established [106–109]. As one miRNA targets multiple transcripts [110], dysfunction of miRNA may result in a wide-scale deregulation of gene expression, often triggering a cascade of events leading to pathogenesis. In 2002, Calin et al. demonstrated* miR-15* and* miR-16* are located at chromosome 13q14 region frequently deleted in B-cell chronic lymphocytic leukemias (B-CLL) [111]. Then, more than half miRNA genes were linked with cancer-associated genomic regions or fragile sites, and their amplification or deletion in human cancers supported miRNA role in malignant transformation [112]. Since 2005, when Lu et al. classified multiple human cancers, including AML, based on miRNA expression profiles exclusively, and proved general downregulation of miRNAs in tumors compared to normal tissues [51], miRNAs started to be widely investigated in cancers and other diseases.

MicroRNAs were also described as regulators of mammalian hematopoiesis [113, 114]. In 2004, three microRNAs, which modulate mouse hematopoietic lineage differentiation, were found by Chen et al. [113]:* miR-181*, a positive regulator of B-lymphoid cell differentiation,* miR-223*, nearly exclusively expressed in BM and myeloid cells, and* miR-142*, found at highest levels in B-lymphoid and myeloid lineages. Georgantas et al. identified 33 miRNAs specifically expressed in CD34+ hematopoietic stem-progenitor cells (HSPCs) [115]. The identified miRNA signature included* miRNA-17, -24, -146, -155, -128*, and* -181*, holding early hematopoietic cells at a stem-progenitor stage and blocking their maturation,* miRNA-16, -103*, and* -107* responsible for block differentiation of later progenitor cells, and* miRNA-221*,* -222*, and* -223* controlling terminal stages of hematopoietic differentiation. Some miRNAs indeed presented lineage-specific expression, which suggested the limitation of function to e.g., lymphoid (*miRNA-146*), erythroid (*miRNA-221* and* -222*), or granulocytic (*miRNA-223*) development. Inhibition of erythropoiesis and erythroleukemic cell growth through* KIT* gene suppression by* miR-221* and* -222* was also reported by Felli et al. [116] whereas granulopoiesis regulation by a minicircuitry involving* miR-223*,* NFIA* and* CEBPA*, by Fazi et al. [117]. Other miRNAs were able to control different processes, e.g., myelopoiesis and erythropoiesis, as* miRNA-155* [115]. Contrary to the results of Chen et al. [113] who studied hematopoiesis on murine model,* miR-142* expression was not detected in human hematopoietic cells by Georgantas et al. [115]. Later, Bissels et al. deepened the knowledge about miRNA-regulated hematopoiesis by combining analyses of microRNA and mRNA profiles in CD133+ and CD34+ hematopoietic stem and progenitor cells [118]. In both types of cells, 25 highest expressed miRNAs accounted for 73-74% of the total miRNA pool. However, the most abundant miRNAs were rather common for both progenitor cell types, except for* miR-142-3p*, which was upregulated in CD34+ cells, to the level of up to 5,000 copies per cell. Remarkably, one of* miR-142-3p* targets seemed to be* CD133* gene. The authors found 18 miRNAs expressed differentially between the CD133+ (ancestral) and CD34+/CD133- (later progenitor) cells.* miR-10a, -99a, -125a* and* b*, and* miR-146a* and* b*, expressed at highest level in CD133+ cells, were postulated to maintain the stem-cell character, whereas* miR-484* and other miRNAs upregulated in CD34+ cells, probably blocked cell differentiation at a later stage. Generally, differentially expressed miRNAs were involved in inhibition of differentiation, prevention of apoptosis, and cytoskeletal remodeling.

In 2007, Mi et al. [54] showed that discrimination of AML from ALL is possible through miRNA expression profiling. Among 27 miRNAs differentially expressed between AML and ALL, four were sufficient to distinguish these two types of acute leukemia:* let-7b* and* miR-223* were significantly upregulated in AML whereas* miR-128a* and* miR-128b* were downregulated in AML comparing to ALL [54]. In 2010, Wang et al. [119] conducted similar research on Chinese cohort of 85 patients and found 16 miRNAs differentially expressed between AML and ALL. Half of them were previously reported by Mi et al. [54] (*miR-23a, miR-27a/b, miR-128a, miR-128b, miR-221, miR-222, miR-223*, and* let-7b*), but eight have not identified previously in this context (*miR-17, miR-20a, miR-29a/c, miR-29b, miR-146a, miR-150, miR-155*, and* miR-196b*). In addition, prognostically relevant signatures were determined for ALL, AML and non-M3-AML. One miRNA,* miR-146a*, was strongly inversely correlated with OS of both acute leukemias in two independent patient cohorts.

Garzon et al. studied miRNA expression in APL cells treated with all-trans-retinoic acid (ATRA) [120], and found upregulation of* miR-15a, -15b, -16-1, let-7a-3, let-7c, let-7d*,* miR-223, -342*, and* -107*, and downregulation of* miR-181b*. The observed ATRA modulation of* NFIA, RAS* and* BCL2* gene expression corresponded with the fact the mentioned genes were targets of* miR-107, let-7a* and* miR-15a/miR-16-1*, respectively. Then, Garzon et al. [121] evaluated the miRNA expression in 122 newly diagnosed AML cases comparing to* CD34*+ cells from 10 healthy donors, and found 26 differentially expressed miRNAs, all downregulated in AML, e.g.,* miR-126, -130a, -135, -93, -146, -106b*, and* -125a*. Expression level of some miRNAs was variable within AML and correlated with AML cytogenetics, prognosis and clinical features. For example,* miR-181* was downregulated particularly in AML with multilineage dysplasia whereas* miR-155* and* miR-181b* positively correlated with WBC (white blood cell count). In AML with balanced 11q23 translocations, many tumor suppressor miRNAs, targeting known oncogenes, were downregulated, e.g.,* miR-34b *(targeting* CDK4* and* CCNE2*),* miR-15a* (targeting* BCL2*),* let-7* family (targeting* RAS* genes), and* miR-196* (targeting* HOX* genes). In trisomy 8, only upregulated miRNAs were identified, including those located at chromosome 8, e.g.,* miR-124a* whose known target is* CEBPA*. NK-AML was the most heterogeneous; therefore the identified miRNA signature was not predictive of NK-AML. However, five miRNAs overexpressed in AML (*miR-199a and b, miR-191, miR-25*, and* miR-20a*) were associated with adverse patient outcome.

Debernardi et al. [122] showed strong correlation of* miR-181a* expression with the AML FAB subtypes (elevated in M1 and M2), and with the expression of its predicted targets. Half of them, e.g.,* BCL2L11, KLF3, MAP2K1*, were negatively correlated with* miR-181a* expression. Havelange et al. [123] observed two other mRNA-miRNA interactions: negative correlation between* miR-181a* and* miR-181b*,* miR-155*, and* miR-146* expression with that of genes involved in immunity and inflammation (*IRF7 *and* TLR4*), and positive correlation between* miR-23a, miR-26a, miR-128a*, and* miR-145* expression level with that of proapoptotic genes (*BIM *and* PTEN*). Association of the last three miRNA with apoptosis was experimentally validated. Lineage-associations were showed for* miR-23a* and* miR-196a* (positive correlation with myeloid differentiation),* miR-191*,* miR-222* and* miR-17* (negative correlation with erythroid differentiation). Interaction analysis induced the authors to conclude that a small group of miRNAs coordinately regulates protein-coding transcriptome influencing the same group of genes (presumably the key players) within the pathway.

In 2008, distinctive patterns of miRNA expression associated with cytogenetic and genetic AML subtypes were determined by Dixon-McIver et al.[124], Jongen-Lavrencic et al. [125], and Li et al. [126]. Dixon-McIver et al. [124] measured the expression level of 157 miRNAs in 100 AML patients and two AML cell lines, with the use of bead-based flow cytometric miRNA expression assay, and found 33 miRNAs with differential expression level between AML and normal BM, 17 upregulated (*let-7e, miR-27a, -30d, -142-5p, -155, -181a, -181b, -181c, -195, -221, -222, -324-5p, - 326, -328, -331, -340, -374*), and 16 downregulated (*miR-*9^*∗*^*, -15b, -26a, -30a-3p, -34c, -103, -147, -151, -182, -184, -199a, -302*b^*∗*^*, -302d, -325, -367, -372*). Moreover, they associated t(15;17) translocation with upregulation of miRNAs located in the 14q32 imprinted domain, e.g.,* miR-127, miR-154, miR-15*4^*∗*^*, miR-299, miR-323, miR-368*, and* miR-370*. In AML with inv(16), high level of* miR-99a, miR-100*, and* miR-224* expression, was observed whereas t(8;21) AML presented high expression of* miR-146a* and a decrease of* miR-133a*. High degree of variability across samples was noted for* miR-10a* and* miR-125b*. Jongen-Lavrencic et al. [125] found a set of strongly upregulated microRNAs (*miR-382, -134, -376a, -127, -299–5p*, and* -323*) in t(15;17), partially overlapping with the APL signature defined by Dixon-McIver et al. [124]. Clustering of AML cases with miRNA expression revealed that inv(16) were sometimes mixed with t(8;21) and shared a part of miRNA signature, which is not unexpected as these both AML subtypes belong to CBF AMLs. Predictors of most AML subclasses, containing from a few to several dozen miRNAs, were built for AML with* NPM1* mutation, and even for AML with* FLT3*-ITD or* FLT3*-TKD mutations which were not separated from other AMLs as a result of global miRNA expression-based clustering.

Li et al. [126] found miRNA signatures composed of 2-24 miRNAs able to distinguish AML with* KMT2A* rearrangement, t(15;17), t(8;21) plus inv(16), t(8;21), inv(16), and normal controls. They noted that* miR-126/12*6^*∗*^ were specifically overexpressed in both t(8;21) and inv(16) AMLs, while* miR-224, miR-368*, and* miR-382* in t(15;17). In* KMT2A*-AML, significant overexpression of miRNAs from polycistronic miRNA cluster,* mir-17-92*, was observed. A minimal class-predictor contained only seven miRNAs:* miR-126, -12*6^*∗*^*, -224, -368, -382, 17-5p*, and* -20a*. Interestingly, differential expression of* miR-126/12*6^*∗*^ was not associated with DNA duplication nor mutation, but probably resulted from epigenetic regulation. Gain- and loss-of-function experiments revealed that high expression of* miR-126* inhibits apoptosis and increases cell viability and proliferation, synergistically with the fusion gene* RUNX1*-*RUNX1T1*. From 674 predicted* miR-126* targets, the authors empirically tested 12 genes and confirmed that only one of them,* PLK2*, was indeed regulated by* miR-126*.

MicroRNA expression pattern correspondence with FAB classification was shown by Wang et al. [127] who noted that M1, M2, M3 and M4 tended to depart from each other more effectively than M5. Apart from miRNAs reported previously, the authors identified a spectrum of new miRNAs whose expression strongly correlated with particular AML FAB types, e.g.,* miR-1300, miR-1180, miR-297, miR-610* and* miR-650* overexpressed exclusively in AML M1. High expression of some miRNAs was common in a few FAB types, e.g.,* miR-181a-d, miR-221* and* miR-222* by M1, M2 and M3. The most distinct miRNA expression pattern was shown in AML M3, with 36 miRNAs strongly and exclusively upregulated, e.g.,* miR-370, miR-224, miR-382, miR-154* described earlier, and* miR-100, miR-195, miR-452, miR-654-3p* not reported previously. Comparing to normal PBMCs, all AML samples displayed downregulation of* miR-29a* and* miR-142-3p* which were proposed by the authors as AML diagnostic biomarkers.

Analysis of miRNA expression data in CN-AML, performed by Marcucci et al. [128] allowed identification of miRNA signature of prognostic relevance. Upregulation of* miR-181a* and* b* was associated with low risk whereas overexpression of* miR-124, -128-1, -194, 219-5p, 220a, and -320* with increased risk of failure to achieve CR, relapse or death. Increased miRNA levels were correlated with increased expression of genes involved in innate immunity, encoding toll-like receptors, interleukins and caspases. Some of them were putative targets of* miR-181*.

## 13. Mutation-Defined AML Subtypes

Progressive accumulation of transcriptomic data regarding both mRNA and miRNA expression allowed more precisely characterize AMLs with the recurrent mutations.

### 13.1. AML with Mutated* CEBPA*

Valk et al. [61] first tried to determine gene expression profiles specific for AML with particular mutations. Having a set of nearly 300 samples, the authors were able to distinguish a unique gene expression signature for AML with* CEBPA* mutations.* CEBPA* gene, mutated in 5% to 15% of all AML cases encodes a critical regulator of hematopoietic stem-cell maintenance and myeloid differentiation, therefore unique gene expression pattern was not unexpected for samples with the loss-of-function* CEBPA* mutation. Valk et al. [61] showed that the most prominent features of* CEBPA*-mutated AML were* CD7* overexpression and downregulation of* CTNNA1, TUBB* and* NDFIP1*. Interestingly, hierarchical clustering of all AML samples segregated AMLs with* CEBPA* mutations into 2 different clusters, and one of them included also samples without any known mutations or chromosomal aberrations. In their subsequent paper, the authors, using bisulfite genomic sequencing, revealed that this previously unidentified subset of AML was represented by samples where* CEBPA* gene promoter was hypermethylated [129]. In fact, within this mysterious AML cluster identified by Valk et al. [61],* CEBPA* levels were very high in AML cases with* CEBPA* mutations whereas in AML with wt variant,* CEBPA* expression was minimal or undetectable, due to its epigenetic silencing [129]. Detailed characteristics of* CEBPA*-silenced AML samples showed that they expressed both, myeloid markers (*CD13, CD33, *and* MPO*), and T-lymphoid markers (e.g.,* CD7* mentioned above). In addition, high expression of the myeloid oncogene* TRIB2* and* NOTCH1* gene, encoding a membrane receptor and transcriptional regulator of T-cell development, was noted. In a part of those AML patients, activating* NOTCH1* mutation was identified. Moreover,* TRIB2* was determined to be a direct target of* NOTCH1* signaling. Later, the authors found that* CEBPA* methylation was accompanied by aberrant hypermethylation of many genes compared to* CEBPA*-mutated AMLs or with normal* CD34*+ hematopoietic progenitor cells [130]. This could explain an observed* in vitro* decreased response of* CEBPA*-silenced AML to myeloid growth factors and makes this AML subtype susceptible to dynamically developing treatment with demethylating agents. Interestingly, comparison of genome-wide methylation pattern with GEP revealed only a minimal overlap (12 unique genes, including* CEBPA*) between the differentially expressed and differentially methylated genes. This suggested that gene expression and genome methylation are biologically independent processes.

### 13.2. AML with Mutated* NPM1* and the Paradox of HOX Gene Expression

Since the time a 4-nucleotide insertion in* NPM1* gene and its significance in AML was discovered by Falini et al. in 2005 [52], much attention was paid to unveiling the mechanism of AML triggered by* NPM1* mutation.* NPM1* encodes a multifunctional protein, involved in ribosome biosynthesis and transport, DNA replication and repair, chromatin remodeling, protein chaperoning, regulation of cell cycle, embryogenesis and oncogenesis [131, 132]. Although* NPM1* protein localizes mainly in the nucleolus, it constantly shuttles between nucleus and cytoplasm. The 4-nucleotide insertion in the last exon of* NPM1* gene results in aberrant cytoplasmic accumulation of a protein and, consequently, affects its functions.* NPM1* gene is mutated in about 30% of AML and in 50% to 60% cases of adult NK-AML. Given that NPM-cytoplasmic positive (NPMc+) AML reveals unique molecular and clinical features [133], it was introduced into WHO classification as a separate entity [13]. An invaluable contribution to cognition of AML promotion by* NPM1* mutation was made by GEP.

Alcalay et al. [134] first claimed NPMc+ AML represented a distinct entity, which can be easily distinguished from other AML samples, regardless of the karyotype. They compared global gene expression of 58 AML NPMc+ samples with prevalence of NK-AML and frequent occurrence of* FLT3* mutations, to the group of 20 NK-AMLs without* NPM1* mutations and lower occurrence of* FLT3* mutations. Unsupervised approach showed* NPM1* mutation status was the strongest clustering parameter. A selected 369-gene-predictor efficiently segregated NPMc+ from NPMc- patients. Interestingly,* NPM1* transcript level did not differ between these two groups, indicating the lack of* NPM1* mutation influence on* NPM1* gene expression. I confirmed this observation by analysis of* NPM1* alternative transcripts with droplet digital PCR (ddPCR) [135]. In the NPMc+ patients,* CD34* and* CD133* antigens, as well as* POU4F1* and* CDKN2C*, were suppressed whereas a number of homeodomain-containing transcription factors, including* HOX* and* TALE* genes, were activated [134]. Because several* HOX* genes are highly expressed in HSCs and their expression decreases within cell differentiation, the authors concluded* HOX* gene activation is a mechanism of stem-cell phenotype maintenance utilized by AML blasts. The obtained results explained the overexpression of* HOX* genes described earlier in an uncharacterized subgroup of NK-AML samples [57], which probably in significant proportion carried* NPM1* mutation.

A miRNA-based expression signature of AML with mutated* NPM1* was later published by Garzon et al. [136]. The signature consisted of upregulated* miR-10a* and* b*, members of* let-7* and* miR-29* families, and* miR-15a-16-1* and* miR-17-18a-19a-20a* clusters. Contrary,* miR-204* and* miR-128a*, predicted to target* HOX* genes, were downregulated in* NPM1*-mutated AML, what is consistent with the observed upregulation of* HOX* genes in this AML subtype. The authors proved positive correlation between* miR-10a* and* HOXB4* expression, and confirmed that* miR-204* targets* HOXA10* and* MEIS1* genes. Similarly, other authors showed three miRNAs located in intergenic regions in the* HOX* clusters,* miR-10a, miR-10b*, and* miR-196a-1*, were highly positively correlated with* HOX* gene expression [122, 123]. Jongen-Lavrencic et al. [125] observed in AML with* NPM1* mutation not only overexpression of* miR-10a* and* miR-10b*, but also overexpression of* miR-196a* and* miR-196b*.

From the other side, Verhaak et al. [137] found that* HOX*-gene-based discriminative signature was not limited to AML with mutated* NPM1*. They showed* HOX*-based classification produced high percentage of false positive results, including AML cases with 11q23 abnormalities and* KMT2A* gene rearrangements, which corresponded with the results described above. One of possible explanations is the fact that* NPM1* mutation in AML is not exclusive. More insight into* HOX* gene phenomenon was given by Andreeff et al. [71], who measured, using real-time RT-PCR technique, expression of 39* HOX* genes in 115 de novo AMLs representing various cytogenetic types. While in normal CD34+ cells homogeneous expression of* HOX* genes was observed, AML samples were very heterogeneous in the matter of* HOX* expression. As previously reported, low levels of* HOXA* and* HOXB* expression was noted in favorable cytogenetic AMLs. Overexpression of* HOX* genes was detected in AMLs with intermediate cytogenetics and in AMLs with* NPM1* mutation, usually associated with favorable prognosis. Considering impact of* FLT3*-ITD, the authors observed higher* HOX* expression in AML samples with both mutations,* NPM1* and* FLT3*-ITD, than in AML with exclusive* FLT3*-ITD.

Biological significance of* NPM1* mutation with concomitant* FLT3*-ITD, and* NPM1* mutation with concomitant NRAS mutation, was also verified with the use of mouse knock-in models [138]. Overexpression of HOX genes, enhanced self-renewal, expansion of hematopoietic progenitors, and myeloid differentiation bias, were common for both combinations, which indicated the persistence of transcriptional signature specific for* NPM1* mutation in hematopoietic progenitors of both double-mutants. Comparing to wt mice, dramatically altered gene expression profile was only observed in* NPM1*-*FLT3*-ITD mutants which also had higher leukocyte counts, early depletion of common lymphoid progenitors, and a monocytic bias, presenting more acute course of the disease.* NPM1* and Nras-mutants, characterized by granulocytic bias, developed AML with a longer latency and a more mature phenotype. Moreover, additional somatic mutations were required for AML progression. The molecular-level results, including GEP, underpinned the higher frequency and significantly worse prognosis of AML with simultaneous* NPM1* and* FLT3*-ITD mutations.

Kühn et al. [139] explained the phenomenon of* HOX* and* FLT3* gene upregulation in* NPM1*-mutated AML as a result of the activity of chromatin regulators,* KMT2A* and* DOT1L*. Earlier, KMT2A and DOT1L methyltransferases were known to positively regulate* HOX* gene expression in normal hematopoiesis and AML with* KMT2A* rearrangements [140]. Many* KMT2A* fusion partners interact with DOT1L. With the use of CRISPR-Cas9 genome editing, small-molecule inhibition and RNA-seq, the authors proved that KMT2A and DOT1L control the expression of* HOX*,* MEIS1* and* FLT3* (which is a downstream target of MEIS1), and also cell differentiation in* NPM1*-mutated leukemia, despite the lack of* KMT2A* rearrangement [139]. Interaction of wt KMT2A with another protein, called menin, and their association with* HOX* and* MEIS1* promoters, were required for* HOX, MEIS1*, and* FLT3* upregulation. DOT1L showed a synergistic effect. Combinatorial inhibition of the menin-KMT2A interaction and DOT1L more profoundly suppressed* HOX*,* MEIS1*, and* FLT3* expression, and induced differentiation of* NPM1*-mutated AML. Therefore, novel and possibly less toxic therapeutic strategies emerged for the acute leukemias with* NPM1* mutation and concomitant* FLT3*-ITD.

### 13.3. AML with* FLT3*-ITD

*FLT3* gene, encoding FMS-like tyrosine kinase 3, is mutated in one-third of AML patients. Usually, the consequence is constitutive activation of the kinase receptor what impairs hematopoietic cell signaling and disturbs hematopoiesis. The presence of* FLT3*-ITD without concomitant* NPM1* mutation is well-established marker of poor AML prognosis. In contrast to AML with mutated* NPM1* or* CEBPA*, gene expression signature specific for AML with* FLT3*-ITD was not found for a long time, probably due to the cooccurrence of other mutations. For example, in the study of Valk et al. [61], samples with* FLT3*-ITD were segregated into three clusters. Considering miRNA expression, Garzon et al. [113] reported* miR-155, miR-10a*, and* -10b* were upregulated in AML with* FLT3*-ITD.

Two classifiers, based on the expression of 10 or 34 genes predicting* FLT3*-ITD in* NPM1*-mutated CN-AML were determined by Huang et al. [141] by analysis of two independent AML patient cohorts, each with over 100 CN-AML patients. Among the 6 genes common for both classifiers, one was downregulated (*MIR155HG*,* miR-155* host gene, and noncoding oncogene) and 5 were upregulated, encoding membrane proteins (*TMEM273* and* STON2*), ectonucleotide pyrophosphatase/phosphodiesterase (*ENPP2*), matrix metallopeptidase (*MMP2*), and cytokine signaling suppressor (*SOCS2*).

In 2017, Zhu et al. [142] analyzed four microarray datasets and identified 22 DEGs between* FLT3*-ITD-positive and negative AMLs shared by all four datasets. Reactome pathway analysis revealed correlation of the identified genes with hemopoiesis, hemoglobin metabolic process, hematopoietic or lymphoid organ development, immune system development, and myeloid cell differentiation. Expression levels of* AHSP, EPB42, GYPC* and* HEMGN* genes were negatively correlated with* FLT3* expression. High expression of these four genes in NK-AML with* FLT3*-ITD was associated with better prognosis. In concordance with the fact that* HEMGN* is a direct transcriptional target of* HOXB4*, a negative correlation between* HEMGN* and* HOXB4* expression was found. Based on the data collected, the authors concluded that* FLT3*-ITD might influence AML prognosis by decreasing the expression of* AHSP, EPB42*,* GYPC*, and* HEMGN* genes.

Wellbrock et al. [143] associated* FLT3* mutation with the expression of HH pathway downstream effector, GLI2. Just as the presence of* FLT3* mutation,* GLI2* expression significantly decreased event-free survival (EFS), relapse-free survival (RFS), and OS. Because* GLI2* was coexpressed with* SMO* (Smoothened) and* GLI1*, one can conclude* FLT3* mutation is generally associated with HH pathway activation. In fact, the analysis of an independent patient cohort revealed the expression of* GLI2* and* GLI1*, and* FLT3* mutation could serve as independent risk factors for the survival of AML patients. Interestingly, the expression of three HH pathway ligands, Sonic Hedgehog (*SHH*), Desert Hedgehog (*DHH*), and Indian Hedgehog (*IHH*), undetectable in AML blasts, was detected in primary BM stromal cells. Thus, BM microenvironment seemed to sustain activation of HH pathway, supporting leukemia progression and mediating AML resistance to conventional chemotherapy [143]. Targeting HH pathway emerges as an alternative or complimentary therapeutic strategy against* FLT3*-mutated AML.

### 13.4. AML with* IDH* Mutations

Mutations in* IDH1* and* IDH2* genes, encoding two isoforms of the nicotinamide adenine dinucleotide phosphate (NADP)-dependent isocitrate dehydrogenases, cytosolic and mitochondrial, respectively, occur in 33% of CN-AML patients and confer unfavorable prognosis [144]. Marcucci et al. [144] identified a novel subset of CN-AML with R172 IDH2 mutation, which was mutually exclusive with other known prognostic mutations, associated with lower CR rates and presented distinctive gene and miRNA expression profiles. Comparing to* IDH1/IDH2*-wt patients, AML with R172 IDH2 mutation revealed higher expression of* APP, CXCL12, PAWR, CDC42BPA,* and* SPARC* genes, and decreased expression of* KYNU, SUCLG2, CD93, LY86, LIST1* and* PTHR2*. As far as the above-mentioned overexpressed genes were more or less directly related to AML and cancer, none of the downregulated genes had previously been associated with AML. In the miRNA expression signature specific for R172* IDH2*-AML, members of* miR-125* family (including* miR-125b* which targets the tumor suppressor gene* TP53* and inhibits myeloid differentiation), and two microRNAs not associated with cancer but involved in embryonal stem-cell differentiation,* miR-1* and* miR-133*, were upregulated. None of the downregulated miRNAs (e.g.,* mir-194-1, miR-526, miR-520a-3p*, and* mir-548b*) had been associated with normal hematopoiesis or AML.

### 13.5. AML with* RUNX1* Mutations

Apart from translocations and fusion transcripts, small mutations were also found in runt-related transcription factor 1 (*RUNX1*) gene in 6% [145] to more than 30% of AML patients [146]. In older AML patients, the frequency of* RUNX1* mutation was twice as high as in younger patients [147]. Presence of* RUNX1* mutation was also associated with the resistance to induction chemotherapy [145].

Gaidzik et al. [145] found 148 genes differentially expressed between* RUNX1*-mutated AML and AML with wt* RUNX1*. However, the identified gene expression signature was not exclusive for* RUNX1* mutation but shared with AML with monosomy 7 and MECOM rearrangements, and AML with complex karyotypes, both deprived of* RUNX1* mutation. A key feature of* RUNX1*-mutated AML was deregulation of apoptotic pathway, supported by an increased expression of* BCL2*-like gene,* BCL2L1*.

Association of* RUNX1* mutations with CN-AML poor outcome and distinct gene and miRNA expression was confirmed by Mendler et al. [147]. In older (> 60 years) CN-AML patients with* RUNX1* mutation and wt* NPM1*, genes normally expressed in primitive hematopoietic cells (e.g.,* BAALC, CD109, GNAI1, HGF, * and* FHL1*) and early lymphoid precursors, B-cell progenitors (e.g.,* DNTT, BLNK, FOXO1,* and* FLT3*), were upregulated whereas myelopoiesis promoters, such as* CEBPA*, components of neutrophil granules (*AZU1, MPO*, and* CTSG*), were downregulated. Regarding miRNA profile,* miR-223* and two members of the* let-7* tumor suppressor family were decreased in AML with* RUNX1* mutations. Three other miRNAs, of unknown functions in leukemogenesis,* miR-211, miR-220*, and* miR-595*, were upregulated in* RUNX1*-mutated blasts.

The collected data indicated definitely different biology of* RUNX1*-mutated AML than, for example,* NPM1*-mutated AML, and contributed to the distinction of AML with mutated* RUNX1* as a provisional entity in the revised WHO classification of AML [13].

## 14. AML with Overexpression of Particular Protein-Coding Genes

Valk et al. [61] distinguished a compact cluster of AML samples with overexpression of* MECOM*, transcriptional regulator, and oncoprotein involved in hematopoiesis, apoptosis, development, cell differentiation, and proliferation. High expression of* BAALC* gene, postulated marker of early hematopoietic progenitor cells, was earlier established as an independent poor prognostic factor in CN-AML [42]. Langer et al. [148] proved younger (<60 years) CN-AML patients with* BAALC* overexpression presented distinct gene expression signature, with upregulation of genes earlier associated with poor outcome (e.g.,* HGF, MN1, CD200*), genes involved in drug resistance (e.g.,* ABCB1* alias* MDR1*) and hematopoietic stem-cell markers (*PROM1* alias* CD133*,* CD34*,* KIT*).* CD133 *was the most upregulated gene in high* BAALC* expressers. Interestingly, no differences were found in global microRNA expression, but an inverse correlation between the expression levels of* miR-148a* and* BAALC* was observed suggesting that* miR-148a* might act as a negative regulator for* BAALC*. Later, the same authors focused on the meningioma 1 (*MN1*) gene, encoding a member of gene transcription regulator complex with the nuclear receptor RAR-RXR or the vitamin D receptor [149]. They associated high* MN1* expression with the lack of* NPM1* mutation, increased* BAALC* expression, less extramedullary involvement, and worse outcome. Gene- and microRNA-expression patterns determined from high* MN1* expressers had common features with high* BAALC* expressers (upregulation of* PROM1, CD34, FZD6, CRYGD*,* CD200*, and* ABCB1* genes) and patients with wt* NPM1* (low levels of HOX genes). Positive correlation was also found between the expression of* MN1* gene and the* hsa-miR-126 *family, contributing to proangiogenic activity of VEGF and formation of new blood vessels, and* hsa-miR-424*, which regulates monocyte and macrophage differentiation. Apoptosis-related* hsa-miR-16* and miRNAs involved in malignant transformation (e.g.,* hsa-miR-19a* and* hsa-miR-20a* members of the* miR-17-92* polycistron), as well as* hsa-miR-100* and* hsa-miR-196a*, were downregulated in AML samples with higher* MN1* expression.

Metzeler et al. [150] who analyzed the expression levels of* ERG, BAALC* and* MN1* in over 200 CN-AML patients with the use of oligonucleotide microarrays, confirmed the association of high level of expression of the studied genes with inferior OS and a lower rate of CR. Indeed, the expression levels of all three genes were highly correlated. However, in multivariate analyses, high* ERG* expression, similarly as* FLT3*-ITD, seemed to be an independent and strongest predictor of negative prognosis in younger and older CN-AML patients. The results suggested the prognostic value of* ERG, BAALC*, and* MN1* genes might partially overlap, and high* ERG* expression, together with the presence of* FLT3*-ITD, might be a sufficient combination of factors for high-risk stratification in CN-AML.

## 15. Time for Meta-Analyses

After a significant amount of gene expression data had been collected, papers reporting meta-analysis started to appear. For example, in 2010, Miller et al. [151] systematically analyzed the results of 25 AML studies published between 1999 and 2008. In total, close to 16 thousand expression features, corresponding to 5 thousand unique genes, were available from 2,744 patient samples analyzed with 10 different microarray platforms. One-third of genes were reported in more than one study. Several genes, e.g.,* VCAN* and* PGDS*, were identified only in AML cell lines. 25 genes, including 7* HOX* family members,* POU4F1*,* TSPAN7*,* MYH11*,* RUNX1T1*,* RUNX3*,* CD34*, and* MN1*, were reported as AML-specific by at least 8 independent studies.* HOX/TALE* expression was increased in AML with normal cytogenetics,* NPM1* and* FLT3* mutations, and 11q23 abnormalities involving the* KMT2A* gene. Decreased expression of these genes was typical of CD34+ cells, AML with* CEBPA* mutations and AML with cytogenetic aberrations. Considering prognosis-relevant signatures, the authors found only a minority of genes (9.6%) were reported by at least two studies. Among these genes,* BCL11A, TBXAS1, HOXB5, HOXA10, CD34, MN1, NME1, FLT3*, were upregulated whereas genes such as* EML4, C3AR1, SMG1, FOXO1, AZU1*, were downregulated in AML samples with poor prognosis. In AML with* NPM1* mutations, increased expression of* SMC4* gene was reported by 5 different studies. Apart from the selection of genes and pathways shared by different AML studies, meta-analysis made by Miller et al. [151] enabled identifying novel marker genes and potential therapeutic targets which were skipped by single studies. The examples were two genes whose expression correlated with response to therapy, namely* TBXAS1* and* SEMA3F*, increased in AML samples with poor and good prognosis, respectively. This evidently shows that reanalysis of collected transcriptomic data and combining the results from different studies may be an underestimated source of new AML-relevant information.

## 16. Custom-Made Microarrays: An Alternative to Global GEP

Since the time microarray technology was established, multiple types and applications of microarrays were developed [45]. To benefit from this dynamically developing technology, guidelines for microarray gene expression analyses in leukemia were formulated by three European leukemia networks in 2006 [152]. Among all microarray platforms used for gene expression analysis of AML, commercially available GeneChips of Affymetrix™, predominated (see Supplementary Table 1). However, a few prominent AML papers were published based on cDNA microarrays developed at the Stanford University [60, 73] or Lund University [69]. All of the above microarray platforms were generated to study global gene expression. Alternatively, small custom-made microarrays, dedicated to analysis of a selected subset of genes, were harnessed to AML studies. IntelliGene Human Cancer CHIP, cDNA microarray from Takara Biomedicals, as well as two kinds of custom oligonucleotide microarrays, covering 2,304 genes, mainly encoding transcription factors, membrane proteins, growth factors, and proteins involved in redox regulation, were used by Miyazato et al. [91] to identify MDS-specific genes. In-house microarray was applied by Park et al. [153] to study the expression of about 300 prognosis-related genes in 4 clinical AML samples. The genes were selected based on global GEP of AML cell lines and previously published data. Taking advantage of our own experience with custom microarrays, we also designed and generated a boutique microarray dedicated to gene expression analysis of AML [154]. Our AML-array was composed of about 900 oligonucleotide probes complementary to genes selected by the literature search: proven and postulated acute leukemia biomarkers, general oncogenes, genes specifically involved in leukemic transformation, genes related to immune response, and a set of positive (housekeeping human genes) and negative (plant and bacterial) control genes. AML-array was used to analyze gene expression in 33 AML patients without or with minimal maturation (FAB M1 and M2 subtypes) and 15 healthy volunteers (HV). Based on 83-gene classifier, we were able to perfectly distinguish AML from HV samples. The genes overexpressed in AML included well-established AML markers such as* KIT, MYH11, MYC, CEBPA, MN1, MPO, SET*, and* HOXA10*, but also genes rarely discussed in the context of AML pathogenesis, e.g.,* STMN1* (the most discriminative gene in our analysis),* CDK6, ANGPT1* or* ENO1*. The role of genes determined in our analysis as underexpressed in AML, e.g.,* IFITM1, FCN1, S100A9, LTB, LYZ, FCER1G*, was even less clear and demand further research. We found that the upregulation of* CPA3* gene was specific for AML with mutated* NPM1* and* FLT3* genes. Although we observed some gene expression trends, we were not able to find any genes with statistically significant differences between AML subgroups divided according to FAB subtype, mutation status, or response to therapy. This may be due to too small sample size, high homogeneity level within the study group, limited to M1 and M2 FAB subtypes which are not dramatically different, too high technical bias or a preselection of genes which could skip more discriminative genes. Nevertheless, we showed applicability of a small custom array to AML gene expression analysis. Following optimization, it could serve, for example, as a first-line diagnostic tool. With the use of complementary quantitative RT-PCR methods, we identified three genes (*S100A9, ANXA3* and* WT1*) whose expression levels can be used to distinguish between M1 and M2 FAB subtypes. We showed relationship between* STMN1* and* ABL1* expression level, and* FLT3* and* NPM1* mutation status. We have also found correlation between positive response to treatment and high* CAT* expression and low* WT1* expression [154].

## 17. SAGE: Alternative to Microarrays before Massive Sequencing Era

Apart from microarrays, serial analysis of gene expression (SAGE) technique was also applied to AML gene expression profiling [59, 155, 156]. Although this method did not demand prior gene sequence knowledge, produced more quantitative results and was described as very sensitive, it was definitely less common than microarrays and finally was ousted by NGS.

With the use of SAGE, 22 AML samples with four most common translocations, t(8;21), t(15;17), inv(16), and t(9;11), were compared to normal myeloid progenitor cells [155]. Over 2.6 thousand transcripts were abnormally expressed. Altered expression of 56 genes was shared by all AML samples, e.g.,* NUBPL, TRAM2, PTRF* (present* CAVIN1*) (upregulated),* FCN1, LCN2* and* FASN* (downregulated). Other genes were differentially expressed in one or some of the translocations studied. In all translocations except t(8;21), more than 2/3 DEGs were underexpressed. Of note, only a small part of the SAGE results corresponded with the results of published microarray-based experiments. For example, Lee et al. [155] did not observe* MYH11* overexpression in inv(16) nor* RUNX1* and* RUNX1T1* overexpression in t(8;21). In subsequent paper, the authors compared SAGE results obtained for three pooled primary AMLs with t(9;11)(p22;q23) with SAGE-based GEP of Mono Mac 6 (MM6) cell line, representing AML with this particular translocation. Despite generally similar gene expression profile, the authors identified 884 alternatively expressed transcripts corresponding to 83 known genes, mainly related to biosynthetic and metabolic processes. Interestingly,* HRAS* with well-established role in leukemogenesis, and three other genes from ERK1/ERK2 MAPK pathway, governing cell growth, proliferation, differentiation and survival, were overexpressed exclusively in MM6.

## 18. Next Generation Sequencing: Unlimited Perspectives

Microarray boom lasted about 15 years. Since 2006, when a first high-throughput automatic sequencer, Genome Analyzer was launched by Solexa, DNA microarrays were being gradually replaced by the NGS, termed also massive parallel sequencing (MPS). At the beginning, the costs of NGS outbalanced the costs of microarray experiment, but they were soon compensated. While microarrays are still applied for genotyping, due to their simplicity compared to the whole genome sequence analysis, in the field of transcriptome research, NGS is incomparably better. Transcriptome sequencing, called RNA-seq, is able to detect all types of transcripts present in a cell, including noncoding RNAs, products of gene fusions and alternative splicing. In addition, transcriptome sequencing is often combined with a whole genome, exome or targeted resequencing which allows completely characterizing the studied object. In 2013, such comprehensive study of AML was published by The Cancer Genome Atlas Research Network in the New England Journal of Medicine [23]. A total number of 200 cases represented different AML subtypes were sequenced (50 whole genomes and 150 exomes). For the same individuals, analyses of global mRNA and miRNA expression, and DNA methylation were performed. In several cases, RNA-seq revealed increased or exclusive expression of the mutant* DNMT3A*,* RUNX1*,* PHF6,* and* TP53* genes. Gene fusions, including 15 new fusion events with maintained open reading frame, were detected in almost half of AML patients. Hierarchical clustering of gene expression data enabled distinguishing seven AML groups based on mRNA expression and five groups based on miRNA expression. Similarly as in microarray data analysis, the identified groups were highly correlated with AML FAB subtypes, differentiation stage, presence of the recurrent mutations, and patient outcomes. Integration of gene expression and DNA methylation data led to the discovery of a small RNA set within an imprinted locus on chromosome 14. These small RNAs were specifically dysregulated in APL. Patients with* PML-RARA* fusions had generally very distinct mRNA and miRNA signatures that were strongly correlated with each other and with a specific DNA methylation signature. AML with* RUNX1*-*RUNX1T1*, AML with some* KMT2A* fusions, and AML with three mutations (in* NPM1*,* DNMT3A*, and* FLT3*) together were also associated with mRNA and miRNA expression signatures. In compliance with previous research, the most discriminatory miRNAs for the triple-mutant AML were* miR-10a, miR-424, miR-196b, miR-130a*, and* let-7b*. Other transcription factor fusions were correlated with only mRNA expression signatures.

At the end of 2018, functional genomic landscape of acute myeloid leukemia was highlighted by Tyner et al. [25]. The study, within the frame of Beat AML program, included half thousand AML patients, for whom whole-exome sequencing and RNA sequencing data were integrated with the analyses of* ex vivo* drug sensitivity. Clustering of the 2,000 most variably expressed genes allowed to distinguish gene expression signatures associated with genetic and cytogenetic AML groups. Mutations in several genes, e.g.,* TP53* and* ASXL1*, seemed to be responsible for a broad pattern of drug resistance. Specific gene expression signatures were also identified for 78 out of 119 testable drugs. For example, 17 gene expression-based signature predicting sensitivity to ibrutinib was determined. Multivariate modelling was harnessed to estimate contributions of both mutation and gene expression patterns in drug response prediction.

Apart from two most prominent studies mentioned above, a significant number of NGS-based AML papers were published within the last several years. Since referring to all of them is impossible in one review, a few selected examples are highlighted below.

## 19. Better Tools: Better Characterization

Like the microarrays, RNA-seq was used to better characterize particular AML subgroups and cell lines. For example, transcriptome sequencing of three basic myeloid leukemia cell lines, K562, HL-60, and THP1, representing chronic myeloid leukemia (CML), APL and acute monocytic leukemia, respectively, was conducted by Wang et al. [157]. They found ERK/MAPK and JAK-STAT signaling pathways were more highly activated in K562 than in HL-60 cells. Contrary, PI3K/PKB pathway, induced by oncogene* KIT* or* FLT3*, as well as* PML* and* RARA* genes, which are fusion partners in APL, were upregulated in HL-60. Genes related to cell cycle, cell division, and chemokine signaling pathway were also overexpressed in HL-60 cells. Genes upregulated in THP1 cells were enriched in immune defense, inflammatory response, and other processes connected with monocyte functions (e.g.,* LYZ, MPO, HLA-B, IL8*, present* CXCL8, PRG2, SPI1*, former* PU.1*, and* TFRC*). Based on GEP, the authors concluded K562 cells are a good model to study erythroid differentiation, HL-60 cells, chemotaxis and phagocytosis, and THP1, inflammatory response. Gosse et al. [158] described a novel NK-AML cell line, termed CG-SH. A whole genome sequencing revealed the absence of recurrent mutations but novel small alterations were found in several genes, including* GATA2* and* EZH2*. Comparing genome and transcriptome data showed allele-specific expression of* GATA2* gene which resulted from epigenetic silencing. Although the mutation was heterozygous, only a mutated variant was transcribed. Interestingly, genes which are frequently mutated in AML, but not mutated in CG-SH (e.g.,* NPM1*,* GATA2, IDH2*,* RUNX1*, and* TP53*), were upregulated in the studied cell line, however, their levels of expression remained within the ranges observed for 55 AML patients. Differential expression of genes implicated in proliferation, apoptosis and differentiation, was noted for CG-SH cells following cytokine treatment.

Two subtypes of pediatric CBF AML, t(8;21), and inv(16), were compared with the use of RNA-seq by Hsu et al. [159]. Although both CBF leukemias revealed many common features, the authors were able to discover two hundreds of DEGs. In t(8;21) samples, the most upregulated gene was* RUNX1T1*, fusion partner gene, whereas the most underexpressed was* RFX8*. Overexpression of matrix metallopeptidase gene* MMP14* and downregulation of collagen gene* COL23A1 *was typical of inv(16). Compared to NK-AML samples,* HOX* gene family, including* MEIS1* and* NKX2-3* transcription factors, were downregulated in both CBF AMLs. Within NK-AML, two subgroups, with and without* FLT3*-ITD, were not able to distinguish based on GEP. In total, 287 fusion transcripts were identified; 16 of them were novel, including three involving NUP98 gene. In the whole cohort of 64 patients, alternative splicing events (ASEs) differentially expressed across all subtypes were also detected. The predominant alternative splicing events were skipped exon (SE), mutually exclusive exons (MXE) and retained intron (RI).

Singh et al. [160] compared genome-wide DNA binding sites and transcriptome data associated with* RUNX1*-*RUNX1T1*,* CBFB-MYH11*, and* PML-RARA* oncofusion protein expression and found many target genes, pathways, and acetylation patterns are shared between these three fusion transcription factors. In the case of* RUNX1*-*RUNX1T1* and* PML-RARA*, the percentage of common target genes reached 40%. Gene expression analysis revealed both common and unique signatures for each translocation. The unique DEGs included genes described earlier as specific for particular translocation, namely,* TRH, POU4F1, PRAME* and* RUNX1T1* genes for t(8;21),* VCAN, MN1* and* S100A12* for inv(16), and* CTSG* and* PTGDS *for t(15;17). However, even these unique genes were members of the similar pathways, in particular, linked to cell proliferation (e.g., TGFB signaling pathway) and apoptosis. Therefore, the authors hypothesized the three different AML subtypes, despite distinct molecular properties (binding sites; mechanisms of action) exploit common programs of malignant cell transformation.

Eisfeld et al. [161] studied AML with a sole monosomy of chromosome 7 (-7 AML), the most frequent autosomal monosomy associated with poor outcome. In over 30 cases analyzed, the authors not only identified the most frequent AML mutations but found different mRNA and miRNA expression profiles compared to AML with both copies of chromosome 7. Among DEGs, downregulated prevailed, with 94% genes mapping to chromosome 7, affirming dosage effect. The most overexpressed genes were* PTPRM*, encoding a protein tyrosine phosphatase receptor, a regulator of cell growth, differentiation and oncogenic transformation, ID1, a downstream target of oncogenic tyrosine kinases, and* MECOM*, coding for a transcriptional regulator and oncoprotein implicated in hematopoiesis, apoptosis, development, cell differentiation and proliferation. Out of 16 differentially expressed miRNAs, 6 were significantly downregulated, including 5 from chromosome 7 and* miR-9-1* from chromosome 1. Upregulated miRNAs came from two clusters, located on chromosome X (*miR-20b, miR-363,* and* miR-106a*) and chromosome 19 (*miR-99b, miR-125a*, and* miR-let7e*).

RNA-seq data were also collected from 13 patients with deletions of the long arm of chromosome 9 [del(9q)], a rare aberration occurring in about 2% of all AML cases, as a sole abnormality or accompanied by t(8;21), t(15;17), or other cytogenetic aberration [162]. Transcriptome of del(9q), combined with the exome and target amplicon sequencing, was compared with the transcriptomes of 454 AML patients with normal karyotype or various cytogenetic aberrations. Characteristic features of del(9q) AML were mutations in* NPM1*,* DNMT3A,* and* WT1,* more frequent than in other AML subtypes, and downregulation of* TLE4* gene.

Mixed-phenotype acute leukemia (MPAL), a rare type of progenitor leukemia with ambiguous expression of myeloid and lymphoid lineage markers, was studied by Pallavajjala et al. (2018) [163]. RNA-seq combined with whole genomic sequencing (WGS) enabled identifying mutations in 70 genes, different translocations, residing mainly in the noncoding regions of the genome, and describing gene expression profiles in samples from four patients with T/Myeloid MPAL. For two patients with matched diagnostic and remission samples, enriched pathway analysis allowed for association of genes which were upregulated at diagnosis, with pathways involving nucleosome and chromatin assembly and organization.

## 20. Discovery of New Fusion Transcripts

Being a useful tool for fusion transcript detection, RNA-seq was applied to identify a complex 3-way translocation t(8;12;21)(q22;p11;q22) in an individual AML M2 patient [164]. In addition to* RUNX1*–*RUNX1T1* fusion, typical of t(8;21) AML, the patient harbored two additional translocations with the contribution of* VPS13B* gene, a causative gene of Cohen syndrome, encoding vacuolar protein sorting 13, forming* TM7SF3–VPS13B* and* VPS13B–RUNX1* fusion genes.

With the use of a whole transcriptome sequencing, 88 new fusion transcripts were discovered in AML by Wen et al. [165]. In total, 134 fusion transcripts were detected in 45 AML samples, including 29 NK-AMLs. The fusions were predominantly formed between the genes adjacent in the same chromosome, in different orientations, and distributed at different frequencies in the AML cases, regardless of the karyotype. While comparing to other tumors, the authors found only 5 common fusions, all shared with only one tumor type (prostate cancer). It underpins the AML-specificity of the discovered fusions. Out of 114 fusions identified in NK-AML, seven were unique for this AML subtype. Moreover,* CIITA-DEXI* fusion transcript, occurring in three isoforms, was found in 48% of NK-AML cases. Of note, the maximal number of fusion transcripts found in one NK-AML case was 57. Although some fusions were generated posttranscriptionally, these results suggest that genome-level changes are not so rare in AMLs with normal karyotypes. The significance of particular fusions remained to be elucidated.

Also in pediatric CN-AML, novel fusion transcripts were identified with RNA-seq, e.g.,* NUP98-PHF23* [166] or* CBFA2T3-GLIS2* [167, 168], occurring with 2.6% and 4.3-8.4% frequency, respectively. More frequent* CBFA2T3-GLIS2* fusion resulted from a cryptic inversion of chromosome 16 and was correlated with high risk of relapse and poor outcome [167, 168]. Schuback et al. [167] demonstrated the fusion was most prevalent in the youngest patients (<5 years) and absent in adults (>20 years). In another work, Masetti et al. identified another fusion transcript in 40% of the* CBFA2T3-GLIS2*-positive patients [169]. The novel fusion derived from a member of Hedgehog signaling pathway, Desert Hedgehog (*DHH*), and Ras Homologue Enrich in Brain Like 1 (*RHEBL1*) gene, coding for a small GTPase of the Ras family.* DHH-RHEBL1*–positive patients exhibited a specific gene expression pattern, with upregulation of* FLT3*,* BEX1*,* MUC4* and* AFAP1L2* genes. The outcome of these patients was even worse than that of the patients with exclusive* CBFA2T3-GLIS2*-rearrangement. Notably, targeted treatments against AML with* CBFA2T3-GLIS2* are under evaluation. GANT61, the most potent inhibitor of GLI family proteins, which are the final effectors of Hedgehog pathway, seems to be efficient also against GLIS2 chimeric proteins [82].

## 21. Alternative Transcripts: Another Source of Transcriptome Variability

The role of alternative splicing (AS) in AML pathogenesis was first highlighted by Tanaka et al. (1995) [36] who analyzed two of three previously identified alternative isoforms of* AML1* gene, at present termed* RUNX1*. Both transcripts,* AML1a* and* AML1b*, shared a runt homology domain, responsible for DNA binding, whereas transcriptional activation domain was present only in* AML1b*. The authors found that the two AS products regulated hematopoietic myeloid cell differentiation in an antagonistic way, presumably via competing for the binding to* CBF2B* gene encoding transcriptional activator. While* AML1a* inhibited granulocytic differentiation and induced cell proliferation upon granulocyte colony-stimulating factor (G-CSF) treatment, concomitant* AML1b* overexpression recovered the granulocytic differentiation.

Development of NGS contributed to the progress in AS research. Now, the role of splicing abnormalities in AML progression and drug resistance is incontestable [170]. While overexpression of* SRSF1* was associated with solid tumor promotion, mutations in genes encoding splice factors, i.e.,* SF3B1, SRSF2, U2AF1*, are considered as important drivers of hematological disorders such as MDS and AML [170]. Contribution to AML pathogenesis was assigned to splice variants of* FLT3*, aberrant splicing of* BCL2* gene, linked with drug resistance, and overexpression of* WT1* and* E2F1* genes, which encode transcription factors taking part in AS regulation [170]. We recently demonstrated that alternative transcripts of* NPM1* gene are upregulated in AML and ALL at diagnosis, decrease in CR and increase again at relapse [135]. High expression of two* NPM1* gene isoforms was significantly associated with shorter overall and disease-free survival. This suggested that not only mutation but also expression level of* NPM1* gene affects patient outcome. Aberrant proportions of particular* NPM1* splice variants could be linked to abnormal expression of genes encoding alternative splicing factors.

RNA-seq of two samples collected from an individual AML M2 patient at diagnosis and remission were explored in the context of AS by Li et al. (2014) [171] and Gao et al. (2014) [172]. In both studies, a few dozens of differentially splicing events were detected in differentially splicing genes, associated with RNA processing, cellular macromolecule catabolic process and DNA binding.

Shirai et al. (2015) investigated the consequences of the most common* U2AF1* mutation in a transgenic mouse model [173]. Whole transcriptome analysis of hematopoietic progenitor cells of* U2af1-*mutated mice revealed altered hematopoiesis and changes in premRNA splicing. Comparing the results of the analysis with human RNA-seq data from TCGA AML cohort displayed enrichment of* U2AF1*-induced splicing alterations in processing genes, ribosomal genes, and recurrently mutated MDS and AML-associated genes (e.g.,* NPM1*,* BCOR,* and* KMT2D*). The authors concluded sequence-specific AS pattern triggered by mutant* U2AF1* was similar in mouse and human cells.

Li et al. (2018) integrated AS events derived from RNA-seq with H3K79me2 ChIP-seq data across 34 human normal and cancer cell types [174]. Clustering based on skipping exon-associated sites divided all cell types to 6 clusters. Two of them consisted predominately of cell lines derived from hematological malignancies. Moreover, four AML cell lines, mainly with* KMT2A* rearrangements, were found in one cluster, together with one CML cell line. Deregulated genes associated with this particular cluster were involved in mRNA splicing via spliceosome. The obtained results corroborated contribution of epigenetic-mediated splicing events to progression of* KMT2A*-AML and associated alternative splicing mediated by K79me methyltransferase, encoded by* DOT1L* gene, with leukemogenesis.

## 22. Chimeric RNAs: Newly Discovered Contribution to Transcriptome Complexity

Apart from well-established fusions and alternative transcripts, another class of transcripts has been recently discovered in tumor as well as in normal cells by RNA-seq [175, 176]. The new class of functional and potentially oncogenic RNAs, called chimeric RNAs (chRNAs), not only are transcribed from genome regions modified by translocation, inversion, or more complex chromosomal rearrangement, but can be generated as a result of posttranscriptional RNA processing, e.g., cis- or trans-splicing. By combining two or more gene loci, chRNAs also differ from conventional splicing variants. The existence of chRNAs in AML was proved by Ruffle et al. in 2017 [177]. In RNA-seq data from three AML patients, 17 chRNAs were identified, including new* PML-RARA* transcripts with exon junctions not described earlier in t(15;17), and expression changes with time and treatment. Other chRNAs originated from two adjacent genes (e.g.,* VAMP8-VAMP5*), nonadjacent exons separated by thousands of base pairs (e.g.,* SLC16A3-METRNL* and* UBR5-AZIN1*), two distant genes (e.g.,* NONE-CTDP1*), or gene fragments corresponding to opposite chromosome strands. Extending their research to a larger AML patient cohort and normal CD34+ transcriptome, the authors identified four new types of tumor-specific chRNAs recurrently expressed in AML samples (*TRIM28-TRIM28, DHRS7B-TMEM11, PLXNB-BLRD1,* and* SLC16A3-METRNL*). On the basis of* PML-RAR* fusions, it was shown in one patient several isoforms of the same chimeric transcript can coexist and present various sensitivity to therapeutic agents. The resistance to treatment can be indicated by the appearance or increase of fusion transcripts. However, the oncogenic potential of chRNAs needs to be verified by further research.

## 23. Small RNAs from the NGS Perspective

Compared to microarrays, NGS enabled more comprehensive and quantitative analysis of miRNome. Ramsingh et al. [178] analyzed miRNome of one CN-AML patient, female diagnosed as FAB M1, to assess miRNA expression and mutations in miRNA or miRNA binding sites. Small RNA sequencing of leukemic myeloblasts and CD34+ cells pooled from 5 healthy donors revealed expression of 472 miRNAs, including 7 novel miRNAs. The most highly expressed miRNA in both AML and CD34+ cells was* miR-233*. In AML, it represented almost 50% of all miRNA reads. miRNAs, which displayed differential expression between AML and control CD34+ pool, included* miR-362-3p* and* miR-25*, overexpressed and underexpressed in AML, respectively. Comparison of NGS-based miRNA profiling with the array- and RT-PCR-based approaches, showed microarray and real-time analyses underestimated the expression of some miRNA, although the general correlation between platforms was significant. The authors did not find acquired mutations in miRNA genes but revealed several novel germline polymorphisms. Comparing the results of miRNA expression with the sequence of this particular AML patient genome, which was known earlier [21], they identified a single mutation in the putative tumor suppressor gene* TNFAIP2* and proved this mutation generated a new miRNA binding site. As this mutation resulted in a Dicer-dependent translational repression of a reporter gene, the consequence could be a translational repression of* TNFAIP2*, previously described as a target of* PML-RARA* or* PZLF-RARA* fusion genes and highly expressed in hematopoietic cells.* TNFAIP2* mutation was predicted to generate imperfect binding sites for* miR-223* and* miR-181b*, but the experiments conducted on AML cell lines did not confirm contribution of* miR-223* and* miR-181b* and any other known miRNA to the translational repression of mutant* TNFAIP2* 3′-UTR. The authors did not exclude possibility of regulation by a new, unknown yet, miRNA. Nevertheless, the* TNFAIP2* 3′-UTR mutation must be rare as it was not found in any other AML samples from 187 patients screened.

Integrating SNP and mRNA arrays with microRNA profiling of 16 myeloid cell lines, García-Ortí et al. [179] associated expression levels of 19 miRNAs with CNVs affecting their loci. One of these miRNAs,* miR-370*, often upregulated in AML, was proven to target tumor suppressor* NF1*, downregulated in more than 30% AMLs. Because* NF1* suppression activates RAS similarly as RAS-activating mutation, AML patients with* miR-370* overexpression may potentially benefit from additional treatment with either RAS or mTOR inhibitors.

Starczynowski et al. [180] who globally analyzed miRNA localization and expression in human genome using cell line models, discovered that 77% miRNAs mapped to leukemia-associated copy-number alterations, and the expression of only 18% of them was detectable. Furthermore, they found the loss of two selected miRNA,* miR-145* and* miR-146a*, localized in a commonly deleted in AML region 5q, initiated leukemia in mice. Using small RNA sequencing they identified 28 novel miRNAs, 18 of which mapped to leukemia-associated copy-number alterations, and may play a role in leukemogenesis.

Using t(8;21) AML mRNA- and miRNA-sequencing data from TCGA project, Junge et al. [181] constructed a network of 605 transcripts, potential competitors of* RUNX1T1* in miRNA binding. The so called competing endogenous RNAs (ceRNAs) cross-regulate each other by competing for binding to shared miRNAs. The predicted set of ceRNAs contained multiple oncogenes and members of the integrin, cadherin, and Wnt signaling pathways. One-third of those genes were differentially expressed between t(8;21) AML and normal granulocyte-macrophage progenitor cells. Taking into account experimentally validated miRNA binding sites, the authors selected 21 top* RUNX1T1* ceRNAs, including 13 which shared miRNA binding sites with* RUNX1T1*, e.g.,* PLAG1, TCF4, NFIB*, and* YWHAZ*. Therefore, the authors supported the hypothetical miRNA sponge function of* RUNX1T1* gene, particularly its 3'UTR, present in a leukemic* RUNX1*-*RUNX1T1* fusion transcript and overexpressed up to 1000 times in t(8;21) versus other and control samples.

## 24. piRNAs, Extracellular Vesicles, and Transposable Elements

From small RNAs, miRNAs are definitely best characterized and their association with normal and malignant development is well established. Recently, a little longer (25-33 nt) P-element-induced wimpy testis (PIWI)-interacting RNAs (piRNAs), responsible for epigenetic silencing of transposable elements (TEs) in germline tissue, have been correlated with brain functioning and tumor transformation [104, 182]. Aberrant piRNA expression was detected in multiple myeloma and various solid tumors [104]. In cancer cells, piRNAs and PIWI proteins may contribute to tumorigenesis through aberrant DNA methylation leading to genomic silencing and promotion of a “stem-like” state, or, oppositely, through gene expression activation via regulation of histone acetylation and euchromatin formation. Comparative analysis of malignant and normal tissues from 11 organs showed that out of approximately 20,000 piRNA present in human genome, less than 300 are expressed in somatic tissues and more than 500 in corresponding tumors [183]. Although most piRNAs were commonly upregulated across tumors, some piRNAs were expressed in tumor-specific manner. A fraction of small RNAs, abundant in miRNAs and piRNAs, was detected in extracellular vesicles (EVs), secreted by bone marrow mesenchymal stem cells (BM-MSC), which are a component of hematopoietic microenvironment [184]. EVs treatment of hematopoietic stem cells extracted from umbilical cord blood (UCB-CD34+ cells) induced cell survival, suppressed apoptosis and decreased cell differentiation. However, piRNA role in AML remains to be elucidated. Up to date, the only evidence of direct relation between piRNAs and AML pathogenesis was presented by Shiva Bamezai in her Ph.D. thesis, devoted to the role of Argonaute protein PIWIL4 in hematopoiesis and AML [185]. The author showed upregulation of* PIWIL4* gene, encoding one of PIWI proteins, in AML samples, the highest in AML with* KMT2A*-*AF9* translocation conferring poor prognosis. Of note, prognostically favorable AML with* PML-RARA* and inv16 showed the lowest levels of* PIWIL4*. Overexpression of* PIWIL4* was correlated with high expression of genes involved in cell proliferation, such as* FLT3*,* CBL* and* NRAS*. Contrary, depletion of* PIWIL4* in AML with* KMT2A* rearrangements drastically reduced leukemic cell growth* in vitro* and* in vivo*, but did not affect normal cord blood* CD34*+ cell growth. Out of 10 thousand unique piRNAs detected in wt THP-1 cells, over 1000 revealed changed expression following* PIWIL4* knockdown, including 80 mapped to genes indicated by RNA-seq as deregulated by* PIWIL4* depletion. Interestingly, most of these genes belonged to the actin cytoskeleton regulation pathway.

The role of TEs in AML and MDS was highlighted by Colombo et al. [186]. While highly expressed in embryogenesis, TEs are usually methylated and silenced by heterochromatin in the somatic cells. Activation of TEs is being observed in ageing tissues and cancers. In LSCs, which are the most therapy-resistant fraction of AML cells, low expression of TEs was noted, along with the suppression of genes involved in interferon pathway, inflammation, and immune response. Significant suppression of TE expression was also identified in high-risk MDS compared to low-risk MDS. Considering the suppression of TEs in in AML and MDS as a mechanism for immune escape, indicates the potential targets to activate cancer immunogenicity in these myeloproliferative malignancies.

## 25. snoRNA

Small nucleolar RNAs (snoRNAs) are basically involved in the posttranscriptional modification of ribosomal RNAs, in cooperation with protein partners [102, 187]. In recent years, new functions of snoRNAs, which can be submitted to an extensive processing, have been discovered, namely in alternative splicing, regulation of chromatin structure, metabolism, and neoplastic transformation [187]. NGS-based analysis of snoRNAs is more tricky as their size, ranging from 60 to 250 nucleotides [187], overlaps with a gap between conventional small RNA sequencing and RNA-seq, devoted to mRNAs and other RNA molecules longer than 200 nt. Developing their own sequencing approach, Warner et al. [188] showed that snoRNAs, e.g., orphan snoRNAs contained in the imprinted* DLK-DIO3* and* SNURF/SNRPN* loci, are expressed in a lineage- and developmentally restricted manner in human hematopoiesis. Moreover, 120 snoRNAs, including* SNORA21*,* SNORA36C* and* SCARNA15*, displayed consistent differential expression in AML, and, what is even more interesting, all of them were decreased in AML samples compared to normal CD34+ cells. Of note, although the majority of snoRNAs were embedded in the introns of host genes, expression levels of snoRNAs did not correlated with expression or alternative splicing of host genes, which suggested cellular levels of mature snoRNAs were determined by other factors. No somatic mutations were detected in the snoRNA genes, either. By the way, the authors found a few novel snoRNAs and proved standard transcriptome sequencing cannot reliably distinguish unspliced primary host gene RNA from correctly processed snoRNA [188].

## 26. Long Noncoding RNAs

A novel class of functional RNAs, which do not encode proteins and are longer than 200 nt, are referred to as long noncoding RNAs (lncRNAs) [189]. Their role in imprinting and regulation of cell cycle, cell differentiation and apoptosis has been postulated [190]. In 2014, Garzon et al. [191] first reported significance of lncRNAs for AML pathogenesis and prognosis. Using a custom microarray platform, they evaluated lncRNA expression in 148 older CN-AML patients and validated the results in an independent cohort of 71 patients. Distinct lncRNA patterns were determined for AML with* FLT3*-ITD and mutations in such genes as* NPM1*,* CEBPA*,* IDH1*,* IDH2*,* ASXL1*, and* RUNX1*. For example, patients with mutated* NPM1* revealed upregulation of several antisense transcripts of* HOX* genes (*HOXB-AS3*;* MEIS1-AS2*), plasmacytoma variant translocation 1 (*PVT1*), and the coiled-coil domain containing 26 (*CCD26*) lncRNAs. Wilms tumor 1 antisense RNA (*WT1-AS*) lncRNA was found as typical of* FLT3*-ITD signature whereas downregulation of* HOXB-AS3* lncRNA was noted in* CEBPA*-mutated AML.* RUNX1* mutation was associated with the increase of lncRNAs located in the proximity of lymphoid marker genes (e.g.,* BLNK*), the immunoglobulin heavy locus (*IGH*) complex, and vault RNA 1-1 (*VTRNA1-1*), which was linked with multidrug resistance. Of note, no specific lncRNA profiles were found for* DNMT3A* and* TET2* mutations which frequently occur in older CN-AML patients. Instead, prognostic signature composed of 48 lncRNAs was identified, indicating correlation of lncRNA expression with AML treatment response and survival.

In 2017, Schwarzer et al. [192] presented the comprehensive transcriptome landscape of the normal human hematopoietic stem cells and their differentiated progenies. Short and long noncoding RNAs (ncRNAs), together with mRNAs, were characterized with the use of three microarray platforms in 12 distinct cell populations purified with multicolor flow cytometry from blood of healthy donors. The observed cell-type-specific ncRNA expression indicated the tight regulation and coordinated function of this RNA class in human hematopoietic system. Functional analysis of the identified ncRNA fingerprints in the studied cells and in two independent datasets of more than 600 AML samples, revealed 80% overlap of associated gene sets. For example,* HOTAIRM1*, granulocyte-specific lncRNA, was associated with inflammatory and innate immune response pathways, and was strongly correlated with genes upregulated in AML with* NPM1* mutation. In addition, novel ncRNA regulators of granulopoiesis were predicted, e.g.,* LINC00173* expressed specifically in mature granulocytes and negatively associated with the expression of genes related to stemness, cell cycle progression and cancer. The role of* LINC00173* in granulocyte proliferation and differentiation was then confirmed by its transcriptional repression with CRISPR-interference (CRISPRi) in NB4 leukemia cell line. Similarly, gain- and loss-of-function experiments validated the function of miRNAs and lncRNAs of the human* DLK1-DIO3* locus in the differentiation and maintenance of megakaryocytes. Transcriptome analysis of 46 pediatric AML samples allowed identification of prognostically relevant ncRNA signatures shared by normal HSCs and AML blasts of distinct cytogenetic and morphologic subgroups.

In the same year, a systematic analysis of lncRNAs in hematopoiesis and hematological malignancies was also conducted by Delás et al. [193] with a murine model and AML cell lines. First, a catalog of lncRNAs was made, in the 11 types of cells, representing different stages of hematopoietic differentiation and blood cancers. Remarkably, similar expression patterns were observed for protein-coding genes and lncRNAs through hematopoietic system and disease development, which indicated involvement of similar mechanisms of expression regulation. A loss-of-function screen revealed that 20 lncRNAs were required for leukemia progression* in vivo*. Some of them seemed to promote leukemia stem-cell signatures, e.g.,* Pvt1* and* Lilam*, whose functions were correlated with the function of* MYC *oncogene. Another lncRNA, termed* Pilna*, was required for the myeloid lineage during bone marrow reconstitution.

Transcriptome analysis of an individual primary AML sample from TCGA dataset enabled discovering 194 unannotated small RNAs in the 17-35 nt size range, 258 unannotated small RNAs in the range of 36-100 nt, and 977 previously unannotated multiexon lncRNA transcripts [194]. A majority of them were also found in the sequencing data of other AML patients from TCGA collection. Integration of the collected data with RNA-seq data from 179 other AML cases led to identification of a subset of lncRNAs with enriched expression in AML M3 (e.g.,* MEG3*) and other FAB subtypes comparing to normal CD34+ cells. Reanalysis of 200 transcriptomes from TCGA AML dataset was also used to construct prognosis-related lncRNA module pathway network [195]. First, lncRNA coexpression network, composed of 42 functional modules, was generated thanks to integration of data from small and mRNA sequencing. Then, survival analysis was performed for each of the identified lncRNA modules and 8 of them, significantly enriched in 70 pathways (including AML pathway, chemokine signaling pathway and pathway in cancer), appeared to be correlated with patient outcome.

Relation between lncRNA expression and AML prognosis was also studied by Mer et al. [196] who sequenced transcriptomes of 274 intensively treated AML patients from a Swedish cohort and found 33 individual lncRNAs significantly associated with OS. Based on lncRNA expression, the authors classified all AML patients to four distinct molecular subtypes. The reproducibility and prognostic significance of the identified lncRNA-based signatures were validated in an independent AML patient cohort (142 TCGA samples). Remarkably, neither of the lncRNA-driven AML subtypes was found to be highly concordant with any of the conventional clinical or genetic factors, although enrichment in* CEBPA*,* NPM1*,* FLT3*-ITD and* TP53* mutation was noted for particular subtypes. Despite some similarities between lncRNA and mRNA expression, both types of transcripts stratified AML patients in different ways. This suggested a limited overlap in information retrieved from various levels of transcriptome analysis and underpinned the rationale for further lncRNA studies.

In 2019, Bill et al. [197], while analyzing a large set of transcriptome data, collected from 450 CN-AML patients, identified a 111-lncRNA-based signature specific for LSCs. Interestingly, only four out of 111 lncRNAs were downregulated in samples with high stem cell like gene expression profile. The lncRNA signature was mainly composed of long intergenic noncoding RNAs (lincRNAs; 30%), antisense RNAs (19%) and sense intronic RNAs (13%). One of the upregulated lncRNAs in LSCs was* DANCR* (*Differentiation Antagonizing Nonprotein Coding RNA*), lincRNA highly conserved between mice and humans, overexpressed in hepatocellular carcinoma.* DANCR*, described as a regulator of the Wnt pathway, crucial for the biology of LSCs, was proved to play a role in LSC self-renewal and quiescence. Decreased expression of* MYC* and other genes from Wnt pathway in AML cell line after* DANCR* knockdown confirmed association of* DANCR* with Wnt pathway.

## 27. Circular RNA

Though most human and mammalian premRNAs are spliced into linear molecules, the existence of circular RNAs (circRNAs), generated by noncanonical splicing (also termed backsplicing), was sporadically reported within the last 30 years [198]. In leukemia, a spectrum of abnormal* KMT2A* transcripts, including circular isoforms, resulted from exon scrambling was shown by Caldas et al. in 1998 [199]. However, until deep transcriptome sequencing was developed, this phenomenon could not be studied on a large scale. At present, it is known that circRNAs arise from hundreds of human genes in normal and malignant cells [198]. The abundance, diversity, and enhanced stability of circular transcripts in human cells suggest that they not only are a result of splicing side effect, but can play a role in the regulation of important physiological and pathological processes.

You and Conrad who elaborated the acfs algorithm for identification and quantification of circRNAs from single- and paired-ended RNA-seq data [200], demonstrated its efficiency on published AML datasets. Comparing 5 APL and 5 CN-AML cases, the authors found 80 circRNAs with opposite pattern of expression, generated from the host genes crucial to the differentiation and proliferation of myeloid cells. For example,* circ_EMB*, generated from* EMB* gene encoding embigin, transmembrane protein from the immunoglobulin superfamily considered as a cancer biomarker, was highly abundant in APL. Upregulation in CN-AML was noted for* circ_SMARCA5*, originated from* SMARCA5* gene encoding a core component of chromatin remodeling and spacing factor RSF, which promotes cell proliferation. In compliance with the theory of circRNA functioning as miRNA sponges, the authors found* miR-10b* (a member of the* miR-99* family) binding site on* circ_SMARCA5*. As* SMARCA5* expression was shown to be affected by* miR-99*, binding of* miR-10b* by* circ_SMARCA5* could relieve linear* SMARCA5* transcripts from repression and contribute to the accumulation of undifferentiated myeloid cells. Analysis of data from a larger AML cohort revealed that the gene loci frequently mutated in AML produced significantly more circRNAs.

In 2017, circRNAs generated from* NPM1* gene have been extensively studied by Hirsch et al. [201]. In total, in six AML cell lines (including OCI-AML3 line with* NPM1* mutation), one CML cell line, and 3 samples derived from the PBMC fraction of healthy volunteers, 15 circular* NPM1* transcripts were identified, including those previously deposited in circBase and novel ones. Oxford Nanopore technology of long read sequencing was harnessed to verify the internal structure of identified transcripts. One of them (hsa_circ_0075001), which exhibited highly differential expression in the AML cell lines, was quantified in a cohort of 46 AML patients. Based on hsa_circ_0075001 expression, patients were divided into two groups that differed in the expression of more than 2000 other genes. AML patients with high hsa_circ_0075001 expression, presented upregulation of ribosomal protein genes, increase of total* NPM1* expression, and downregulation of genes involved in the Toll-like receptor (TLR) signaling pathway and genes targeted by* miR-181* (e.g.,* CARD8, CASP1, MSR1, SLC11A1, TLR4*), which is deregulated in CN-AML. The expression of hsa_circ_0075001 correlated with total* NPM1* expression, but was not affected by the* NPM1* mutational status. Global analysis of circRNA expression in 10 AML samples and 10 sorted cell fractions form healthy hematopoietic controls evidenced the existence of circRNA transcripts for almost half of all highly expressed genes [201]. Despite a general tendency towards higher circRNA expression from genes with higher parental gene expression, there were some exceptions, e.g.,* circFLT3* expression was not correlated with* FLT3* gene expression in AML samples (though it was in healthy samples). AML patients and healthy controls differed in the expression levels of circRNAs arisen from 27 genes, including* ANGPT1*,* UGCG* and* FLT3*. In addition, AML subgroup-specific circRNA signatures were identified, e.g.,* NPM1*-mutated patients could be distinguished from* NPM1*-wt patients based on their global circRNA expression (but not based on* circNPM1* expression).

L'Abbate et al. [202] who analyzed the architecture and expression pattern of chromosome 8 region with MYC amplification in 23 cases of AML, detected a significant overexpression of* circPVT1*, a circular transcript of* PVT1* gene in the studied AML cases compared to NK-AML. The expression of* circPVT1* was correlated with* PVT1* gene copy-number increase and high* PVT1* expression in AML patients with* MYC *amplification.

Contribution of circRNA to drug resistance was recently shown by Shang et al. with the use of doxorubicin-resistant THP-1 AML cell line (THP-1/ADM) [203]. The authors identified 49 circRNAs differentially expressed between THP-1/ADM and THP-1 cells. One of them,* circPAN3*, was overexpressed not only in THP-1/ADM cells but also in refractory and recurrent AML samples. Silencing of this circRNA restored sensitivity to doxorubicin in THP-1/ADM cells, which indicated its significant role in chemoresistance mediation.

## 28. Focus on Stem Cells

Leukemic blasts which accumulate in BM and PB of AML patients represent cells blocked at a particular stage of differentiation. However, in the majority of GEP studies performed within the first years of microarray technology development, bulk AML cells were compared to the whole pool of mononuclear cells from healthy control samples. Then, the cell sorting techniques were introduced to select particular cell fractions. The example of a study where AML cells were compared not only to unselected healthy BM and PB samples, but also to normal hematopoietic CD34+ cells extracted from BM and PB, was that of Stirewalt et al. [204]. They found 13 genes deregulated in AML compared to all subpopulations of normal hematopoietic cells, including 7 upregulated (*BIK, CCNA1, FUT4, IL3RA, HOMER3, JAG1, WT1*) and 6 downregulated (*ALDHA1A, PELO, PLXNC1, PRUNE, SERPINB9, TRIB2*). Moreover, the expression levels of* WT1, FUT4, CCNA1, HOMER3, JAG1, TRIB2*, and* SERPINB9* were strongly associated with FAB classification whereas* WT1, JAG1, ALDH1A1, TRIB2*, and* PLXNC1* with cytogenetics. For example,* WT1* was the most overexpressed in AML with inv(16) or t(15;17), CCNA1 in t(15;17), while* BIK* expression was absent or extremely low in t(8;21). 7 upregulated genes were also measured in pediatric AML, where* BIK, FUT4*, and* WT1* showed the most significant increase in expression.

After the discovery of the self-renewing leukemic stem cells (LSCs), the main focus was shifted to these early progenitor cells capable of initiating leukemogenesis. How they are different from normal human hematopoietic stem cells (HSCs) was first shown in 2009 by Majeti et al. [205] who identified over 3000 DEGs between normal HSCs and LSCs extracted from AML patients. The selected genes encoded mainly proteins with kinase activity, associated with nucleoplasm, Golgi apparatus, chromosomes, vacuoles and actin cytoskeleton. KEGG pathway analysis revealed the top dysregulated pathways in LSCs were those related to adherens junction, ribosome, regulation of actin cytoskeleton, tight junction, focal adhesion, apoptosis, MAPK signaling, T-cell receptor signaling, pathway, JAK-STAT signaling and Wnt signaling. Some of these pathways were already associated with stem-cell biology and cancer development, other were not studied in cancer stem cells yet. The obtained results emphasized the importance of the LSCs' interaction with their niche in leukemia initiation and progression.

Gentles et al. showed the correlation of high expression of leukemic stem-cell genes with adverse outcomes in AML [206]. Comparing subpopulations of cells extracted from 163 NK-AML samples, the authors identified 52 genes discriminating the LSC-enriched subpopulations (CD34+/CD38-) from the leukemic progenitor cell (LPC)-enriched subpopulations (CD34+/CD38+), among others genes involved in early hematopoiesis, e.g.,* VNN1, RBPMS, SETBP1, GUCY1A3, MEF2C*, and* HOPX*. Genes associated with proliferation, cell cycle, and differentiation were systematically repressed in the LSCs. The identified LSC gene expression signature (LSC score), reflecting self-renewal ability, was validated on four independent datasets of 1047 patients in total, leading to similar conclusions. OS, EFS, and RFS were worse for patients with high LSC score [100].

Ng et al. [207] compared gene expression profiles between 138 LSC+ and 89 LSC- cell fractions from 78 AML patients. From the list of 104 DEGs, 17 most related to stemness were selected to generate a LSC score (LSC17), which could be calculated for each patient as the weighted sum of expression of the 17 genes. Strong association between high LSC17 scores and poor overall and event-free survival was observed, as in the case of higher bone marrow blast percentage at diagnosis, higher incidence of the* FLT3*-ITD and adverse cytogenetics. The LSC score was validated by xenotransplantation assays and by reanalysis of microarray and RNA-seq data from five independent cohorts of more than 900 AML patients with different subtypes. Comparing with other prognostic determinants such as age, WBC count, cytogenetic risk group, and mutational status, LSC17 score was the strongest and independent prognostic factor. In the end, the authors designed a custom NanoString assay to easily analyze expression of 17 genes from the LSC signature. The assay should allow for rapid risk assessment at diagnosis, application of more intensified investigational therapies in the case of high-score patients, and protection of low-score patients against unnecessary toxicity. To test its efficiency in childhood leukemia, Duployez et al. applied the LSC17 score to 228 de novo pediatric AML patients [208]. Indeed, children with low LSC17 score had significantly better outcome (OS and EFS) compared to children with high score. Then, the stemness signature was validated in 257 children from an independent AML cohort. However, prognosis of pediatric patients with low LSC17 was not significantly better than those with intermediate LSC17 score. The differences between adult and pediatric AML patients might result from the different proportion of CBF AML in both groups (twice as high in children). Nevertheless, the authors extended the LSC17 prognostic value to pediatric AML patients, at least with non-CBF AML [208]. Among all negative prognostic factors, including the high LSC17 score, high WBC count, cytogenetic group “other aberrations” and presence of WT1 and* RUNX1* mutations, high LSC17 score gained the best statistics.

Recently, GEP of LSCs, HSCs, and leukemic progenitors from the same AML bone marrow enabled identifying three genes overlapping in the results of two pairwise comparisons (LSCs vs. HSCs and LSCs vs. leukemic progenitors):* S100A8, SOD2*, and* IGFBP7* [209]. Significantly lower expression of* IGFBP7*, encoding insulin-like growth factor-binding protein 7, in LSCs was correlated with reduced sensitivity to chemotherapy. Restoration of* IGFBP7* expression by a recombinant human gene resulted in differentiation, inhibition of LSC survival, and improved response to therapy, without affecting normal hematopoiesis and HSC survival. Therefore,* IGFBP7* gene was postulated to be a factor responsible for the persistence of LSCs.

## 29. Taking Advantage of Model Organisms

Deep insight into the biology of the disease is not possible by the analysis of cells extracted from patient BM or PB.* In vitro* experiments with cell line models are convenient, but have also some limitations. In fact, animal models are the best option to study particular gene function, consequences of early pathogenic events or treatment with potential therapeutics. Using retroviral insertion mutagenesis in mice, Erkeland et al. (2004) [210] identified a number of genes that could be involved in the pathogenesis of AML. In one of their later works, the authors studied the virus integration sites (VISs) and virus common integration sites (CISs) in the human GEP datasets of 285 adult AML samples [61] and 130 pediatric AML samples [68]. First, they noted VIS flanking genes were significantly more differentially expressed between AML clusters than random genes in both datasets. Then the authors identified five regulatory networks involving 110 VIS/CIS genes most differentially expressed in the adult dataset. Network associated with cell growth contained only these genes. Many of them, e.g., interleukin and* STAT* genes, were implicated in cytokine signaling. Another network, consisted of gene expression regulators, was able to discriminate between AML patients with favorable and unfavorable prognosis. In the unfavorable group,* HOXA9*,* MEIS1*, and* CCND3* genes were increased whereas* BCOR* and* GFI1* genes were decreased.

Glass et al. [211] used NGS platform to identify* MECOM* (previously* EVI1*) target genes by comparison in* MECOM*-overexpressing murine myeloid leukemia cell lines (DA-1, NFS-60) before and after shRNA-mediated* MECOM* knockdown.* MECOM*, oncogenic transcription factor associated with human myeloid malignancies of poor prognosis, is overexpressed in 8–10% of adult AML and up to 27% of pediatric leukemias with* KMT2A* rearrangements.* MECOM*-induced leukemic cells present impaired myeloid differentiation, resistance to apoptosis, and aberrant cell cycle regulation, which results in excessive proliferation. Combining RNA-seq expression profiling with ChIP-seq, the authors found MECOM directly bound to and downregulated* Cebpe* gene, encoding a master myeloid differentiation regulator,* Serpinb2* gene, encoding serine protease inhibitor involved in cell cycle regulation, and numerous genes from the Jak-Stat signaling pathway that drive cellular differentiation. In addition, several P2X purinoceptors, responsible for ATP mediated apoptosis in neutrophils and macrophages, appeared to be significantly downregulated in* MECOM* leukemic cells.

An interesting approach was applied by Wilhelm et al. (2011) [212] who generated two related murine leukemic clones through the retroviral overexpression of* Meis*1 and* Hoxa9* genes in the purified fetal liver (FL) cells. Both clones differed in the hematopoietic stem-cell frequency and gene expression profile. Considering microRNAs, only a few miRNAs were really highly expressed; more than 95% of all miRNA transcripts came from the top 15 miRNAs. Functional annotation analysis showed the most differentiating genes between Meis1- and Hoxa9-overexpressing clones were related to immune system development, hemopoietic or lymphoid organ development, hemopoiesis, and myeloid cell differentiation. Both, differential expression and/or differential splicing, were observed in the studied transcriptomes. Moreover, hundreds of single nucleotide variants (SNVs), shared by both cell clones or unique for one of them, were identified with respect to the public reference sequence. The study revealed also a number of unannotated transcribed elements.

## 30. Gene Expression Regulation: New Directions

McKeown et al. (2017) [213] matched the epigenomic circuitry with the transcriptional state of leukemic cells to identify potential new treatment strategies. The authors focused on the large (>20 kb), highly active chromatin regions called “super-enhancers” (SEs) described previously as key oncogenic drivers in tumor cells [214]. Although SEs constitute only about 5% of all enhancers, they are involved in the regulation of the crucial genes defining cell identity and phenotype. 66 AML patients were characterized in terms of enhancers, super-enhancers, gene expression, and mutational status of blasts [213]. On average, 807 SEs per sample with a median length of 22 kb were identified. Most of them were linked to multiple genes. In addition, SE maps from normal HSPCs and monocytes were used to define the signature of differentiation state. The two most pervasive enhancer signatures were found in the genome and named Myeloid Differentiation and HOX Factor Activation, the last correlated with enhancers associated with homeobox (*HOX*) genes,* PBX3*, and* MEIS1*. Based on overall SE distribution, patients were classified to 6 subgroups, including 4 enriched in* NPM1* and* FLT3* mutation, and one containing all AML samples with* KMT2A* translocation. The authors concluded* KMT2A* translocations might induce a unique epigenetic state affecting overall enhancer landscape, which is consistent with the association of* KMT2A* fusion proteins with* DOT1L* deregulation and, consequently, aberrant histone H3K79 methylation. Moreover, patients classified to different SE-clusters showed differences in OS. Discovery of* RARA*-associated SE which differentiated patient samples and correlated with significantly higher* RARA* expression in 25% patients, prompted the authors to test the sensitivity of AML cells presenting a strong SE at the* RARA* locus to RAR*α* targeted compounds. The results of tests conducted on AML cell lines, AML patient derived mouse xenograft models and AML cells* ex vivo*, demonstrated that RAR*α* agonist SY-1425 (tamibarotene) selectively inhibited the proliferation of AML cells with high (but not low)* RARA* expression, stimulating the expression of genes linked to granulocytic differentiation (e.g.,* CD38, ITGAX, ITGAM, *and* CD66*) and retinoic acid response (e.g.,* DHRS3*). In the context of efficiency, comparison of SY-1425 to ATRA, used clinically for APL therapy, showed the prevalence of the tested compound. Transcriptional response of APL to retinoids was similar to the response of AML cell lines with high* RARA* expression to SY-1425. The authors presented the following interpretation of the observed results: while APL-specific PML-RARA fusion protein represses transcription of differentiation-related genes, in non-APL AML,* RARA*-associated SE induces overexpression of unliganded RAR*α* which acts as a transcriptional repressor of genes regulating by RAR*α*. The practical consequence of clinical significance is that some non-APL AML patient may benefit from the ATRA-like therapy, and increased levels of* RARA* mRNA could be used as a prerequisite for the treatment by SY-1425, which has a power to reset RAR*α* transcriptional activity.

Not only genetic and epigenetic factors contribute to AML pathogenesis; initiation, progression and maturation of AML are also affected by posttranslational modification of proteins. The important role of SUMOylation of* sPRDM16* in AML progression was demonstrated by Dong & Chen on leukemic cell lines [215].* PRDM16*, previously termed* MEL1*, encodes transcription factor acting as a H3K9me1 methyltransferase responsible for maintenance of heterochromatin integrity. However, only protein isoform deprived of the histone methyltransferase domain, encoded by a short transcript variant,* sPRDM16*, was associated with AML pathogenesis. The authors showed that SUMOylation of* sPRDM16* changed the expression of genes implicated in wound response, cell proliferation, chemotaxis, differentiation, and cell cycle progression, including 13 genes (e.g.,* KLF10, BCL3, HDAC9, CCL5, IL6R, LIF,* and* NUMB*) involved in proliferation and differentiation of hematopoietic and leukemic cells.

## 31. Single-Cell Sequencing: The Future

Considering AML as a multiclonal cancer, we should be aware of the fact that bulk RNA sequencing reflects what is going on in a dominant clone that is not necessarily a clone of origin. Even application of cell sorting technique may not be sufficient to catch the whole AML heterogeneity as far as we sequence a pool of cells. A promising approach, which can overcome this problem is single-cell gene expression profiling. A pilot single-cell AML study was performed on twenty CD45-positive cells collected from an individual AML patient [216]. GEP showed only 11 out of 20 cells selected for the analysis were putative blasts, i.e.,* CD34*-positive, or HLA-DRA- and CD117-positive. Moreover, two of them were outliers in PCA. Complementary targeted DNA sequencing revealed the presence of mutations in* DNMT3A* and* NPM1*, and* FLT3*-ITD in the analyzed AML sample. However, RNA-seq identified only* DNMT3A* mutation in only one single cell. Possible explanations are not sufficient coverage (although* NPM1* and* FLT3* genes are usually highly expressed in AML), imperfect algorithms for mutation detection in RNA-seq data, and mutational heterogeneity of blast cells. An argument for the last explanation can be found in the report of Shlush et al. [217] who identified in highly purified preleukemic stem cells* DNMT3A* mutation at high allele frequency, but did not detected concomitant* NPM1* mutation. Therefore, the authors concluded* DNMT3A* arose early in AML evolution. The preliminary experiences with single-cell sequencing show this technology demands optimization, but seems to be a strategy of future.

## 32. Commercial Solutions for AML Research and Clinics

As a result of years of transcriptome-scale studies, a few commercial tools dedicated to AML and other hematologic malignancies were developed. Affymetrix technology, which conquered the microarray market, was used by SkylineDx (https://www.skylinedx.com), high-tech commercial-stage biotech company headquartered in Rotterdam, the Netherlands, to design AMLprofiler, a qualitative* in vitro* diagnostic and prognostic microarray supporting the choice of optimal therapy strategy [218]. AMLprofiler combines seven separate assays used for the purpose of cytogenetics, mutation detection, and gene expression analysis, reducing the time between sampling and diagnosis from 4 weeks to 3 days. The time of diagnostic report generation does not exceed 15 min.

Illumina (https://www.illumina.com), the company headquartered in San Diego, California, USA, who practically monopolized the NGS market, released the MiSeqDx system, the first FDA-regulated, CE-IVD-marked, NGS platform for* in vitro* diagnostic testing. Although AML-dedicated kits do not exist for MiSeqDx, the system is customizable and a number of partners collaborates with the company to develop new clinical assays. At present, kits for target sequencing are available for other Illumina systems, e.g., TruSight Myeloid Sequencing Panel, targeting 54 genes, including* CEBPA*,* NPM1* and* FLT3*-ITD, or AmpliSeq for Illumina Myeloid Panel, targeting 40 DNA genes, 29 RNA fusion driver genes, and 5 gene expression levels associated with myeloid cancers, including AML.

Other integrated diagnostic platforms are being developed and validated, e.g., rapid and sensitive NGS-based assay combining karyotyping and mutational screening of AML [219]. Here, three NGS libraries are generated: two DNA-based libraries—for whole genome sequencing and selected variant identification—and one RNA-based library for fusion transcript detection. The whole workflow can be completed within 5 days.

## 33. Conclusions

Within the last two decades, an explosion of AML studies, driven by technological progress, could be observed. AML picture emerging from transcriptome research is very complex and dynamic. AML transcriptome, affected by cytogenetic and genetic variability, resembles a mosaic, composed of many elements interacting with each other. AML subtypes present unique patterns of protein-coding gene and microRNA expression. Moreover, a spectrum of fusion genes, alternative transcripts and newly discovered chimeric RNAs, as well as a lot of noncoding RNAs, including long linear and circular forms, contribute to the complexity of leukemic transcriptome.

Transcriptome data cannot be interpreted separately from genetic and genomic data. Even a sole point mutation can affect expression of numerous genes, e.g., when it occurs in a transcription factor, epigenetic regulator, or a crucial member of a signaling pathway. Therefore, genome, exome, methylome sequencing, or targeted resequencing is often combined with RNA-seq. In some cases transcriptome analyses helped to define which mutation was the driver one. It must be remembered that a disease is not limited to leukemic blasts. Recent studies on BM microenvironment underlined its role in AML initiation, progression, and relapse. Recognition of molecular interplay between LSCs and BM niche not only is necessary to understand the AML biology but also opens novel AML treatment directions.

Many reports proved the power of global transcriptome profiling and proposed application of GEP for AML diagnosis and prognosis. A single microarray-based test usually classified AML subgroups properly without any a priori knowledge. The following studies utilized more advanced generations of microarrays, composed of an increasing number of probes, and included more numerous cohorts of patients. Of note, the results obtained by different groups were largely overlapping. As gene expression-based prediction of the major cytogenetic subgroups was efficient in both, pediatric and adult, AMLs, GEP appears as an attractive alternative to classical cytogenetics which is laborious and time-consuming. On the other hand, there is no need to harness high-throughput technology when simple, e.g., PCR-based, diagnostic tests can easily confirm the presence of the fusion genes.

Because some genes are significantly overexpressed in AML, not only in leukemic blasts but also in BM microenvironment, and their expression affects treatment response, transcript measurement at the time of diagnosis should be obligatory in AML diagnostics. In my opinion, reliable quantitative PCR techniques, e.g., ddPCR, have currently the highest potential to be routinely applied in clinical practice to analyze selected transcript levels. To analyze higher number of transcripts, gene expression arrays or RNA-seq may be applied instead. Although both high-throughput GEP approaches demand specialized, expensive equipment, and well-trained staff, RNA-seq seems to be a superior technology, offering more information at a comparable cost. Within the last few years, small personal, relatively cheap, and portable sequencers started to appear which makes NGS-based tests more available. Moreover, alternative, third-generation sequencing technologies are becoming more and more popular. I believe that future clinical diagnostic laboratories will offer NGS services, not limited to mutation detection, as a standard. Even if accepting GEP as the only diagnostic test would be difficult, it could at least serve as a first screening or a complementary tool.

Summarizing, although gene expression studies were not directly translated into clinical practice up to date, they helped us to understand the biology of tumors and undoubtedly contributed to the improvement of classification of hematological malignancies and risk estimation, which is crucial for optimal treatment decision and directs us towards personalized medicine.

## Figures and Tables

**Table 1:** The list of the genes renamed within the last years and the most commonly used abbreviations.

*Renamed Genes*			

Previous Name	Previous Description	Current Name	Current Description

*AML1*	Acute Myeloid Leukemia 1	*RUNX1*	Runt Related Transcription Factor 1

*Ang-1*	Angiopoietin-1	*ANGPT1*	Angiopoietin 1

*BRN3A*	Brain-3A	*POU4F1*	POU Class 4 Homeobox 1

*CD11c*	CD11c Antigen	*ITGAX*	Integrin Subunit Alpha X

*CD133*	CD133 Antigen	*PROM1*	Prominin 1

*ELA2*	Elastase-2	*ELANE*	Elastase, Neutrophil Expressed

*ETO*	Protein ETO	*RUNX1T1*	RUNX1 Translocation Partner 1

*EVI1*	Ecotropic Viral Integration Site 1	*MECOM*	MDS1 And EVI1 Complex Locus

*FLJ14054*	Homo sapiens cDNA FLJ14054 fis, clone HEMBB1000240	*NPR3*	Natriuretic Peptide Receptor 3

*IL8*	Interleukin-8	*CXCL8*	C-X-C Motif Chemokine Ligand 8

*MADH1*	Mothers Against Decapentaplegic Homolog 1	*SMAD1*	SMAD Family Member 1

*MDR1*	Multidrug Resistance Protein 1	*ABCB1*	ATP Binding Cassette Subfamily B Member 1

*MEL1*	MDS1/EVI1-Like Gene 1	*PRDM16*	PR/SET Domain 16

*MLL*	Mixed Lineage Leukemia	*KMT2A*	Lysine Methyltransferase 2A

*MLLT2*	Myeloid/Lymphoid Or Mixed-Lineage Leukemia (Trithorax (Drosophila) Homolog); Translocated To, 2	*AFF1*	AF4/FMR2 Family Member 1

*MLLT4*	Myeloid/Lymphoid Or Mixed-Lineage Leukemia Translocated To, 4	*AFDN*	Afadin, Adherens Junction Formation Factor

*NICAL*	NEDD9-Interacting Protein With Calponin Homology And LIM Domains	*MICAL1*	Microtubule Associated Monooxygenase, Calponin And LIM Domain Containing 1

*OPN*	Osteopontin	*SPP1*	Secreted Phosphoprotein 1

*PTRF*	Polymerase I And Transcript Release Factor	*CAVIN1*	Caveolae Associated Protein 1

*PU.1*	Hematopoietic Transcription Factor PU.1	*SPI1*	Spi-1 Proto-Oncogene

*Abbreviations*			

AML	acute myeloid leukemia		

ALL	acute lymphoblastic leukemia		

APL	acute promyelocytic leukemia		

BM	bone marrow		

CBF	core binding factor		

circRNAs	circular RNAs		

CLL	chronic lymphocytic leukemia		

CML	chronic myeloid leukemia		

CN-AML	cytogenetically normal AML		

CR	complete remission		

DEGs	differentially expressed genes		

DFS	disease-free survival		

*FLT3*-ITD	*FLT3*-internal tandem duplication		

FAB	French-American-British (classification system)		

GEP	gene expression profiling		

HH	Hedgehog		

HSCs	hematopoietic stem cells		

HSPCs	hematopoietic stem-progenitor cells		

lncRNAs	long noncoding RNAs		

LSCs	leukemic stem cells		

MDS	myelodysplastic syndromes		

MRD	minimal residual disease		

MSC	mesenchymal stem cells		

NGS	next generation sequencing		

NK-AML	normal karyotype AML		

NPMc+	NPM-cytoplasmic positive		

OS	overall survival		

PB	peripheral blood		

PBMCs	peripheral blood mononuclear cells		

SAGE	serial analysis of gene expression		

snoRNAs	small nucleolar RNAs		

TCGA	the Cancer Genome Atlas		

TEs	transposable elements		

WBC	white blood cell		

WHO	World Health Organization		

## Supplementary Materials

Supplementary Materials Supplementary Table 1 presents the results of PubMed search demonstrating a great effort of the scientific community put into acute myeloid leukemia research. All terms used for searching are included, along with the corresponding numbers of papers found, with two additional filters applied. Part of the information presented in Supplementary Table 1 was used to generate Figure 1 plots. Supplementary Table 2 lists all AML transcriptome papers cited in the manuscript. The following information is included: title, first, and last author, laboratory, name of the journal, year of publication, AML sample size, sample description, and technology applied. All papers are divided according to the technology (microarray/PCR or NGS) and then ordered chronologically.
