# Supplementary material for: Not Only Mutations Matter: Molecular Picture of Acute Myeloid Leukemia Emerging from Transcriptome Studies
Source: J Oncol. 2019 Jul 30;2019:7239206. doi: 10.1155/2019/7239206 (PMC6699387; doi:10.1155/2019/7239206)
Supplement: Supplementary Materials — Supplementary Table 1 presents the results of PubMed search demonstrating a great effort of the scientific community put into acute myeloid leukemia research. All terms used for searching are included, along with the corresponding numbers of papers found, with two additional filters applied. Part of the information presented in Supplementary Table 1 was used to generate Figure 1 plots. Supplementary Table 2 lists all AML transcriptome papers cited in the manuscript. The following information is included: title, first, and last author, laboratory, name of the journal, year of publication, AML sample size, sample description, and technology applied. All papers are divided according to the technology (microarray/PCR or NGS) and then ordered chronologically. [file 7239206.f1.zip › mat.7239206.v1 (1).docx]

**Supplementary Table 1.** PubMed search results.

| Searched term | Total number of paper | Number of reviews |
| --- | --- | --- |
| (acute myeloid leukemia) | 64,916 (29,434) | 6,944 (3,951) |
| (acute myeloid leukemia) AND microarray | 613 (378) | 34 (25) |
| (acute myeloid leukemia) AND transcriptome | 332 (132) | 18 (8) |
| (acute myeloid leukemia) AND gene | 17,977 (6,426) | 2,045 (764) |
| (acute myeloid leukemia) AND protein | 31,588 (4,293) | 2,950 (429) |
| (acute myeloid leukemia) AND expression | 16,110 (7,844) | 1,247 (598) |
| (acute myeloid leukemia) AND (gene expression) | 10,533 (3,415) | 912 (306) |
| (acute myeloid leukemia) AND (gene expression) AND microarray | 493 (251) | 24 (18) |
| (acute myeloid leukemia) AND (gene expression) AND (sequencing) | 503 (241) | 50 (25) |
| (acute myeloid leukemia) AND (gene expression) AND (next generation sequencing) | 109 (33) | 23 (11) |
| (acute myeloid leukemia) AND (gene expression) AND (RNA-seq OR RNAseq OR RNA seq) | 67 (37) | 2 (1) |
| (acute myeloid leukemia) AND (microRNA OR miRNA) | 735 (438) | 93 (53) |
| (acute myeloid leukemia) AND (non-coding RNA OR noncoding RNA) | 1384 (115) | 82 (11) |
| (acute myeloid leukemia) AND (piRNA OR piwi-interacting RNA) | 562 (0) | 7 (0) |
| (acute myeloid leukemia) AND (circRNA OR circular RNA) | 22 (13) | 0 (0) |
| (acute myeloid leukemia) AND genome | 7,045 (661) | 621 (119) |
| (acute myeloid leukemia) AND (genome sequencing) | 749 (188) | 72 (46) |
| (acute myeloid leukemia) AND (whole genome) | 325 (143) | 36 (29) |
| (acute myeloid leukemia) AND exome | 176 (133) | 22 (17) |
| (acute myeloid leukemia) AND (exome sequencing) | 164 (129) | 22 (17) |
| (acute myeloid leukemia) AND (alternative splicing) | 267 (76) | 14 (3) |
| (acute myeloid leukemia) AND (stem cells) | 8,396 (3,067) | 1,213 (593) |
| transcriptome | 59,497 (42,523) | 4,106 (3,159) |
| microRNA OR miRNA | 88,369 (66,546) | 14,246 (9,325) |
| piRNA OR piwi-interacting RNA | 72,574 (1,315) | 3,068 (261) |
| circRNA OR circular RNA | 8,614 (4971) | 644 (515) |
| next generation sequencing | 45,862 (29,321) | 5,993 (4908) |
| microarray | 87,526 (82,160) | 4,972 (4,520) |
| leukemia | 271,365 (177,763) | 28,622 (18,958) |
| breast cancer | 343,449 (262,799) | 40,946 (33,370) |
| lung cancer | 266,473 (159,843) | 29,295 (20,515) |
| cancer | 3,229,599 (1,399,625) | 414,493 (223,403) |

Two additional filters were applied for search: (1) Language: English; (2) Search fields: Title/Abstract (numbers in parentheses). The date of search: 25.04.2019. The number of reviews is included in the total number of papers. The numbers highlighted in colors were used to generate plots (three different colors correspond with three different plots from Figure 1: plot A - yellow; plot B – green; plot C- blue).
